# Multi-biofunctional graphene oxide-enhanced poly-*L*-lactic acid composite nanofiber scaffolds for ovarian function recovery of transplanted-tissue

**DOI:** 10.1038/s41536-022-00236-5

**Published:** 2022-09-16

**Authors:** Liang Yan, Lingjuan Wang, Jiachen Wu, Yuanzheng Wu, Xianyu Zhu, Qiaojuan Mei, Yinhua Song, Yang Liu, Ling Zhang, Jihui Ai, Kezhen Li, Guangming Qing, Yong Zhang, Xianjin Xiao, Yuliang Zhao, Wenpei Xiang

**Affiliations:** 1 Institute of Reproductive Health, Tongji Medical College, Huazhong University of Science and Technology, Wuhan, Hubei 430030 China; 2 CAS Key Laboratory for Biomedical Effects of Nanomaterials and Nanosafety, Institute of High Energy Physics and National Center for Nanoscience and Technology, Chinese Academy of Sciences, Beijing, 100049 China; 3 GBA Research Innovation Institute for Nanotechnology, Guangdong, 510700 China; 4 Department of Obstetrics and Gynecology, Tongji Hospital, Tongji Medical College, Huazhong University of Science and Technology, Wuhan, Hubei 430030 China; 5 Hunan Jialin Biotechnology Corporation, Changsha, 410000 China; 6 Department of Hepatobiliary Surgery, Union Hospital, Tongji Medical College, Huazhong University of Science and Technology, Wuhan, Hubei 430030 China; 7 University of Chinese Academy of Sciences, Beijing, 100049 China

**Keywords:** Regenerative medicine, Biomedical materials

## Abstract

In this study, we successfully constructed the new graphene oxide/poly-*L*-lactic acid (GO/PLLA) nanofiber scaffolds with a hydrophilic surface and porous network structure that were highly favorable for cell infiltration. When employed these new nanofiber scaffolds for a wide range of tissue engineering applications, it was expected to promote graft tissue survival and angiogenesis. The new GO/PLLA nanofiber scaffold with an appropriate concentration of 1.0 wt% was applied for the restoration of ovarian function and reserve in mice with primary ovarian insufficiency (POI). After co-transplanting the normal ovarian cortex loaded on these new nanomaterials into the in situ ovarian tissue of POI mice, the fusion of transplanted ovarian cortex with damaged ovarian tissue was improved, as well as the ovarian function and the follicle numbers. Moreover, angiogenesis was observed clearly and proved to exist in the transplanted tissue and nanomaterials, with the most conspicuous effect after co-transplantation with 1.0 wt% GO/PLLA nanofiber scaffold. In addition, nitric oxide (NO) production by phosphorylated endothelial nitric oxide synthase (p-eNOS) in vivo was proven to be involved in the effect of GO and PLLA on the improved survival rate of the transplanted ovarian cortex. This study provides a new method for the fertility preservation of ovarian tissue cryopreservation and transplantation, as well as a new strategy for the transplantation of other organs.

## Introduction

Innovative technologies, involved in early diagnosis, precision radiotherapy, neoadjuvant chemotherapy and so on have significantly improved the 5-year and long-term survival rate of cancer patients during childhood or in childbearing age^1–3^. However, irreversible damage to female ovaries caused by radiotherapy, chemotherapy drugs and ionizing radiation could even lead to the permanent loss of fertility^4–7^. Ovarian tissue cryopreservation (OTC) and transplantation (OTT) have been developed as the only options for fertility preservation in prepubertal patients with cancer and are also suitable for young women with hormone-sensitive tumors or time-limited malignant invasive tumors^8–11^. Autotransplantation of cryopreserved/thawed ovarian cortex, regarded as the experimental technique and an important method of fertility preservation, has become the focus of research in human tissue engineering^5,6,12^. Livebirth after reimplantation of the cryopreserved ovarian cortex was first reported in Lancet in 2004^13^. By the end of 2017, more than 130 babies had been born worldwide using OTT technology^14^. However, there were still some unanswered questions about OTC followed by OTT despite the obtained pregnancy outcomes: ischemia-reperfusion injury occurs after the reimplantation of resuscitating ovarian tissue, and the lack of oxygen further leads to a significant reduction in the primordial follicle population before the establishment of blood supply, which is another biggest factor affecting the survival of ovarian tissue after transplantation^15–17^. Evidence from animal experiments suggested that complete ovarian transplantation with arteriovenous anastomosis could shorten the time of ischemic injury before revascularization and restore ovarian function in the long term, but multiple factors (e.g., the lack of appropriate cryoprotectant, the complexity of vascular anastomosis operation, etc.) made it difficult to implement in clinical applications^18,19^. Some studies have shown that tissue perfusion could be accelerated by the co-transplantation of ovarian tissue and exogenous endothelial cells (ExECs), but a co-transplantation system related to biomaterials has not been reported thus far^20^.

It has been reported that the large specific surface area of GO and its abundant oxygen functional groups (such as epoxy, hydroxyl, and carboxyl groups) can promote the specific microenvironment interaction of cells and tissues, activate intracellular signaling, and promote cell proliferation, differentiation and angiogenesis^21–24^. Therefore, the combination of GO and biomaterials to build an in vivo angiogenic composite material is expected to provide a new auxiliary means for organ transplantation. It has important scientific significance and broad application prospects. These biomaterials have the characteristics of biocompatibility and degradability and play an important role in the fields of surgery, tissue engineering and regenerative medicine^25,26^. Among them, poly-*L*-lactic acid (PLLA) has been widely used in clinical diagnosis and treatment^27,28^. Its biocompatibility and degradability are excellent, which makes it one of the most recognized biomaterials.

However, we still face two key scientific issues. First, although the angiogenic effect of GO had been proven, promoting tissue fusion and improving organ transplantation in vivo involved a more complex life process, and thus far, the effect of GO in vivo had not been seen in any previous research report. Second, the most widely used PLLA had the disadvantages of low initial strength and fast strength decay, and the process of tissue fusion and regeneration was slow and required certain mechanical support. Therefore, we needed to modify PLLA to enhance its mechanical properties without affecting its performance and biocompatibility.

Fortunately, as a two-dimensional hexagonal nanosheet, GO exhibits unique electrical and mechanical properties and has been widely used as an effective enhancer to enhance the mechanical properties of various polymers^29,30^. Therefore, combining GO with PLLA could subtly strengthen the shortcomings and endow PLLA with new functions. Herein, we first mixed GO nanosheets into the PLLA matrix and then used electrospinning technology to prepare nanofiber scaffolds with the required porous structure to meet the controllable mechanical requirements and support tissue fusion and regeneration. Taking ovarian tissue transplantation as a model, we used this material to connect the transplanted ovarian tissue and the original ovarian tissue in mice. The ovarian tissue co-transplanted with the material easily survived, and there was obvious angiogenesis between the implanted ovarian tissue and the material. We reported for the first time that graphene meets the improvement of nanomaterials in organ transplantation. We anticipated that GO/PLLA nanofiber scaffolds will serve as a very useful auxiliary material and play an important role in the field of organ transplantation.

## Results

### Preparation and characterization of GO/PLLA nanofiber scaffolds

**Fig. 1:**
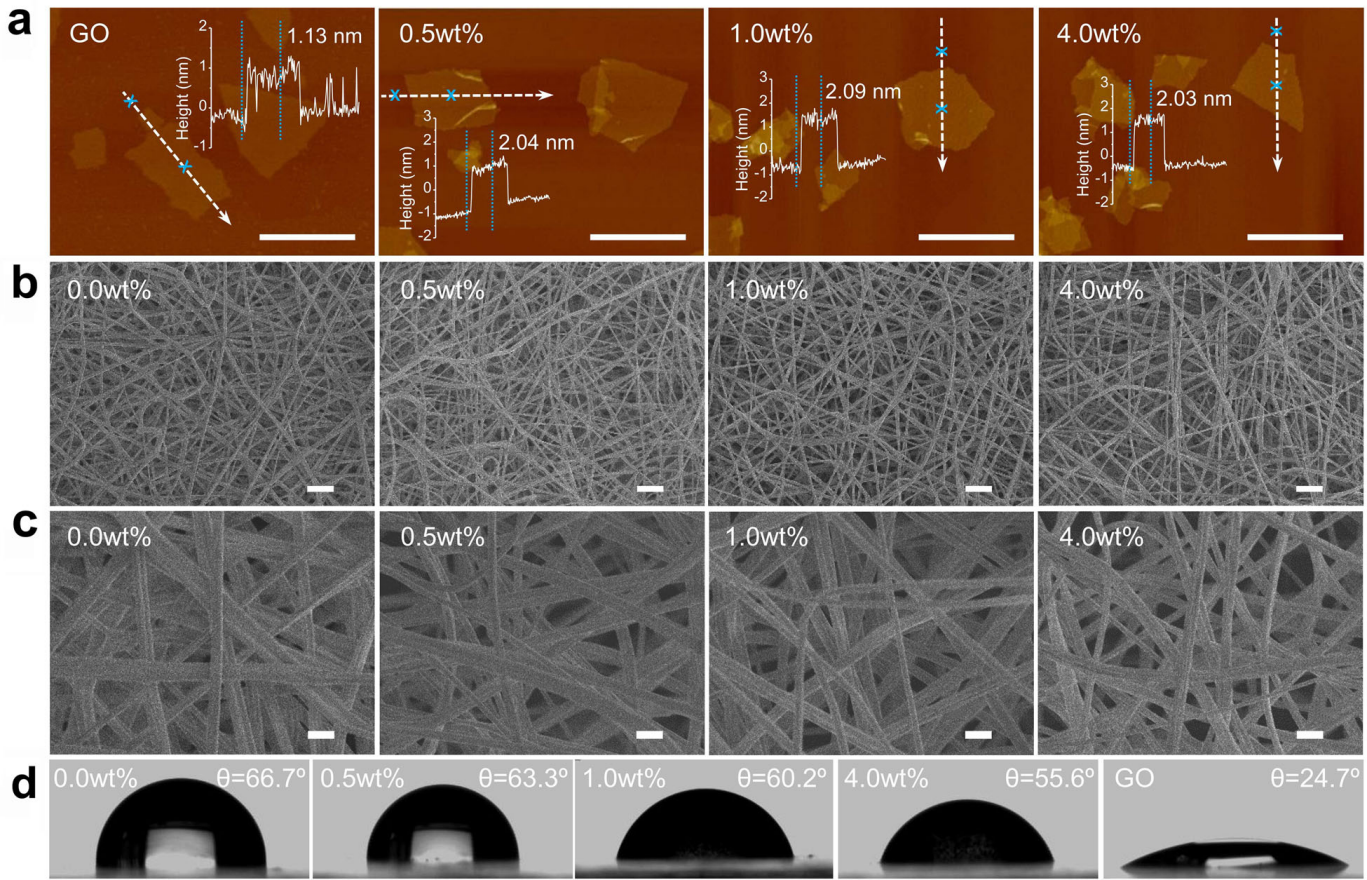
Characterization of GO/PLLA nanofiber scaffolds. **a** AFM images of GO nanosheets interacting with or without PLLA. **b**, **c** SEM images of GO/PLLA nanofiber scaffolds. **d** Contact angle images of GO/PLLA nanofiber scaffolds.

In terms of chemical structure, GO nanosheets could be regarded as an amphiphilic two-dimensional material with an edge-to-center distribution of hydrophobic and hydrophilic domains, the co-occurrence of which was demonstrated by X-ray photoelectron spectroscopy (XPS) analysis (Supplementary Fig. 1), endowing GO nanosheets with novel liquid phase properties, particularly their ability to adsorb molecules at the interfaces. Atomic force microscopy (AFM) images showed that GO nanosheets were fully exfoliated in water with a thickness of ~1 nm, in good agreement with the reported literature (Fig. 1a)^31^. Upon mixing, almost no significant change in the lateral size of GO nanosheets was observed, and graphene nanosheets maintained a flat morphology, while their thickness increased to ~2 nm, and the surface roughness increased up to ~0.451 nm, independent on the concentration of GO (Supplementary Figs. 2, 3a, b), suggesting the dispersive attachment of a large density of PLLA on the nanosheet surface. Given that PLLA had nonpolar and hydrophobic groups, attractive hydrophobic interactions between GO nanosheets and PLLA occurred in the hydrophobic domain of GO nanosheets. Notably, no remarkable sedimentation or phase separation was present during the mixing process, demonstrating that GO nanosheets adequately localized into the two phases of dichloromethane (DCM) and N,N-dimethylformamide (DMF) to interact with PLLA to form GO/PLLA nanofiber scaffolds.

Then, GO/PLLA nanofiber scaffolds with various concentrations of GO were prepared via the general procedure depicted in Supplementary Fig. 4. During this process, PLLA solution with a concentration of 10 wt% was first made using DCM/DMF (1:1, v/v). The reason for selecting such a solution system was because (i) DCM was a good solvent for the complete dissolution of PLLA, and (ii) DMF, a poor PLLA solvent with a high boiling point, could effectively avoid the continuous needle clogging resulting from polymer crystallization, which was caused by the quick evaporation of DCM. As shown in the optical images of the as-made scaffolds, their color gradually became dark with the increase in the concentration of GO, suggesting that GO was successfully incorporated into PLLA matrix (Supplementary Fig. 5). The Raman spectra of nanofiber scaffolds reflected the presence of strong interfacial interactions in the nanofiber scaffolds, illustrating the absorption of PLLA onto the graphene surface as well (Supplementary Fig. 6). Compared to the pure PLLA nanofiber scaffold, SEM images of GO/PLLA nanofiber scaffolds indicated continuous, uniform, and defect-less nanofibers with smooth surfaces and that no GO nanosheets protruded from the nanofiber surface (Fig. 1b, c). This indicated the strong interfacial interaction between GO nanosheets and PLLA, facilitating substantial improvement of the mechanical performance of PLLA matrix (Supplementary Fig. 7). Moreover, the average diameter of nanofibers gradually decreased with increasing GO concentration, indicating that GO had an influence on the nanofiber size (Supplementary Fig. 8). The water contact angle of the nanofiber scaffolds changed from 66.7° to 55.6° with increasing GO concentration, suggesting that the incorporation of GO into PLLA matrix made the surface more hydrophilic (Fig. 1d and Supplementary Fig. 9a). The porosity of these nanofiber scaffolds was relatively high, ranging from 0.81 to 0.84 (Supplementary Fig. 9b). The hydrophilic surface and the porous network structure of GO/PLLA nanofiber scaffolds made it possible to be highly favorable towards cell infiltration and after implantation, thereby making them suitable for a wide range of tissue engineering applications.

### Degradation behavior of GO/PLLA nanofiber scaffolds

The degradation behavior of GO/PLLA nanofiber scaffolds in aqueous and physiological environments was systematically evaluated to match the tissue requirements. After immersion of nanofiber scaffolds in water and PBS, no significant changes in the morphology or microstructure were observed with increasing degradation time, demonstrating that all of these nanofiber scaffolds had a negligible degradation rate during the 28 days (Supplementary Figs. 10, 11). In the case of Dulbecco’s modified Eagle’s medium (DMEM) and DMEM supplemented with FBS, there was no clear degradation of the pure PLLA nanofiber scaffold during the first several days, while its surface became rough over a period of 14–28 days, and the well-formed and dense PLLA nanofibers gradually swelled, subsequently undergoing deformation and partial deterioration (Supplementary Figs. 12, 13). This was likely because it took some time for ions as well as proteins to diffuse and then permeate into the surface of nanofibers and the phase interfaces. As degradation continued, the increases in the accessible surfaces allowed the penetration of ions and proteins into the denser phase of nanofibers, finally resulting in their dramatic degradation. After 28 days, the characteristic peaks in the Raman spectra could not be detected (Supplementary Figs. 14–17), and the weight loss of the PLLA nanofiber scaffolds reached 7.06% in DMEM and 9.77% in DMEM/FBS (Supplementary Fig. 18). Notably, for GO/PLLA nanofiber scaffolds, several degradation phenomena, including swelling and merged nanofibers, were observed up to 3 weeks. These changes were analogous to those observed for pure PLLA nanofibers but less dynamic and dependent on the GO concentration; however, variations in the morphology and structures were not proven. These results suggested that GO nanosheets were able to improve the barrier properties of PLLA matrix, thereby enabling control of the degradation rate without changing other properties. Furthermore, these GO/PLLA nanofiber scaffolds displayed similar degradation behavior after transplantation into the body (Supplementary Fig. 19).

### Cytotoxicity assessment of GO nanosheets and optimization of GO concentration in GO/PLLA nanofiber scaffolds for culturing granulosa cells extracted from ovaries

**Fig. 2:**
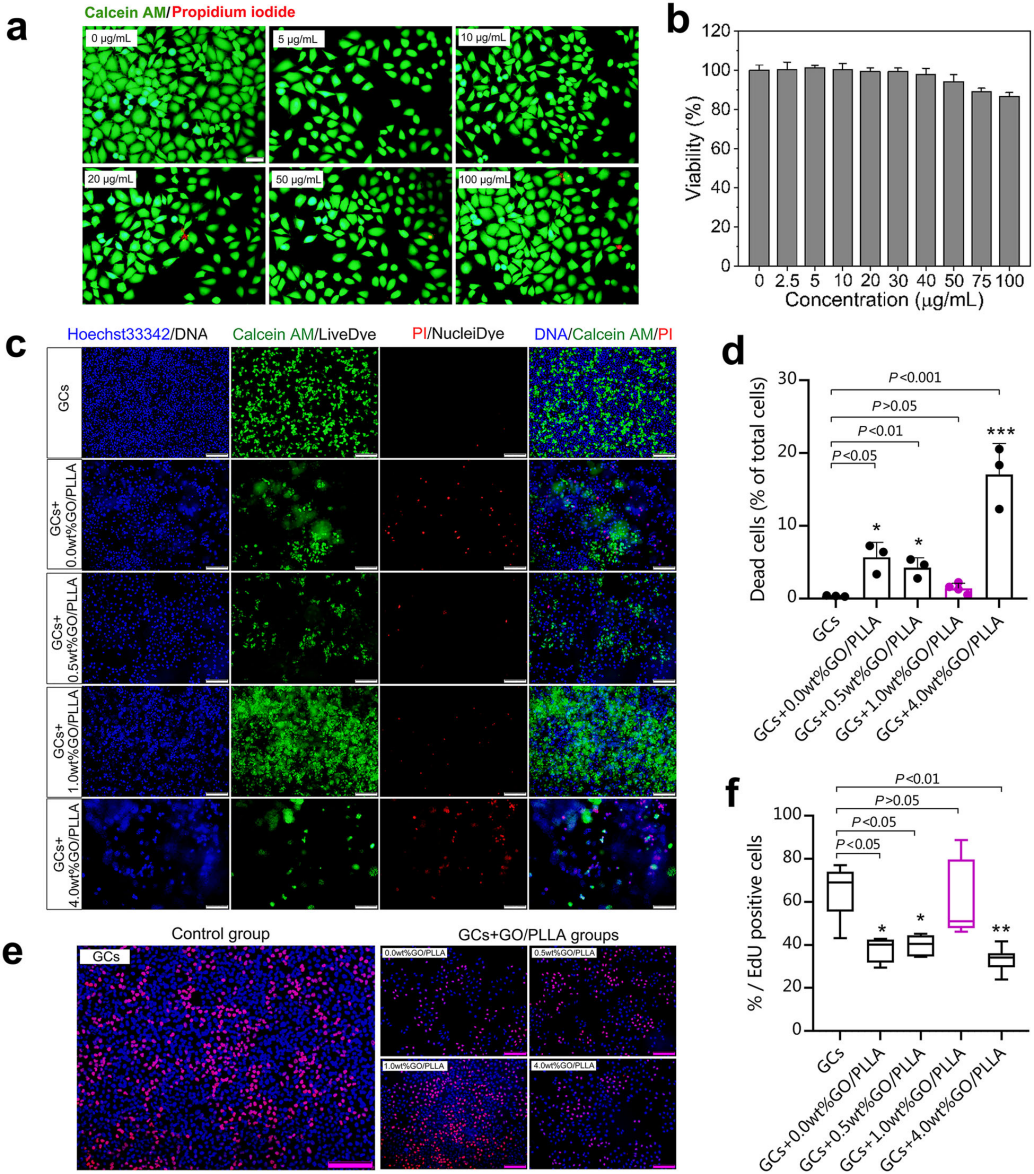
Cytotoxicity assessment of GO nanosheets and the optimization of GO concentration in GO/PLLA nanofiber scaffolds. **a** The most representative image of Live-Dead staining of GCs with GO nanosheets at various concentrations. Scale bar: 50 μm. **b** Cell viability of GCs treated with GO nanosheets at various concentrations. **c** Live-Dead fluorescence staining images of GCs polymerized with different GO/PLLA nanofiber scaffolds. Scale bar: 200 μm. Blue: nucleus; Green: cytoplasmic orthoesterase activity (live cells); Red: damaged cell membranes allow ethidium homodimer to bind to DNA (dead cells). **d** The dead cells ratio (% of the total cells) after culturing for 72 h. **e** The most representative images of the EdU assay of GCs with GO nanosheets at various concentrations. Scale bar: 200 μm. **f** The percentage of EdU-positive cells after culturing for 72 h. Statistical significance levels are set at *P < 0.05, **P < 0.01, ***P < 0.001 by one-way ANOVA (Multiple comparisons). Data are presented as mean ± SD.

Calcein AM/propidium iodide (CA/PI) staining showed an extremely low percentage of dead cells with GO nanosheets at various concentrations (Fig. 2a), as well as with no significant effect on cell viability (Fig. 2b), suggesting minimal cytotoxicity of GO nanosheets on granulosa cells (GCs). Granulosa cells were then harvested and planted on the nanofiber scaffolds with various concentrations of GO (0.0, 0.5, 1.0, and 4.0 wt%) for three days compared to the GCs group (referred to as the control group), a conspicuous increase of dead cells percentage was uncovered in the other three groups except for the cells planted on GO/PLLA nanofiber scaffold with 1.0 wt% GO (abbreviating the group as “GCs+1.0 wt%GO/PLLA”). Moreover, Live-Dead staining showed that the percentage of dead cells in GCs+1.0 wt%GO/PLLA group was significantly lower than that in other three groups (GCs+0.0 wt%GO/PLLA, GCs+0.5 wt%GO/PLLA, GCs+4.0 wt%GO/PLLA) (Fig. 2c, d). Furthermore, the EdU assay showed that cell proliferation in the GCs+1.0 wt% GO/PLLA group was significantly improved compared with other three groups (0.0 wt%, 0.5 wt%, 4.0 wt%) (Fig. 2e, f). Moreover, a remarkably higher percentage of EdU-positive cells was also found in the control group than those in the GCs+0.0 wt%, GCs+0.5 wt%, and GCs+4.0 wt% GO/PLLA groups, and no significant differences were found when compared with the GCs+1.0 wt% GO/PLLA group (Fig. 2e, f).

### Construction of the mouse model for POI

To establish POI models in mice, they were treated with cisplatin (1.5 mg/kg) for ten days by intraperitoneal injection in succession. After one month of cisplatin administration, atrophied ovaries appeared with severe stromal hyperplasia (Supplementary Fig. 20a), and reduced follicles at all developing stages were different from those in the control group (Supplementary Fig. 20b). In comparison, the results showed that cisplatin could significantly decrease the body weight of mice and reduce the weight index (ovarian/body) (Supplementary Fig. 20c). In addition, when compared with the control group, low levels of E_2_ and AMH were observed in the POI group, as well as markedly elevated gonadotropin levels (such as FSH and LH) (Supplementary Table 1). All of the above results indicated the successful establishment of POI models in mice.

### Co-transplantation of ovarian tissue loaded on 1.0 wt% GO/PLLA nanofiber scaffold accelerates the survival of ovarian tissue

**Fig. 3:**
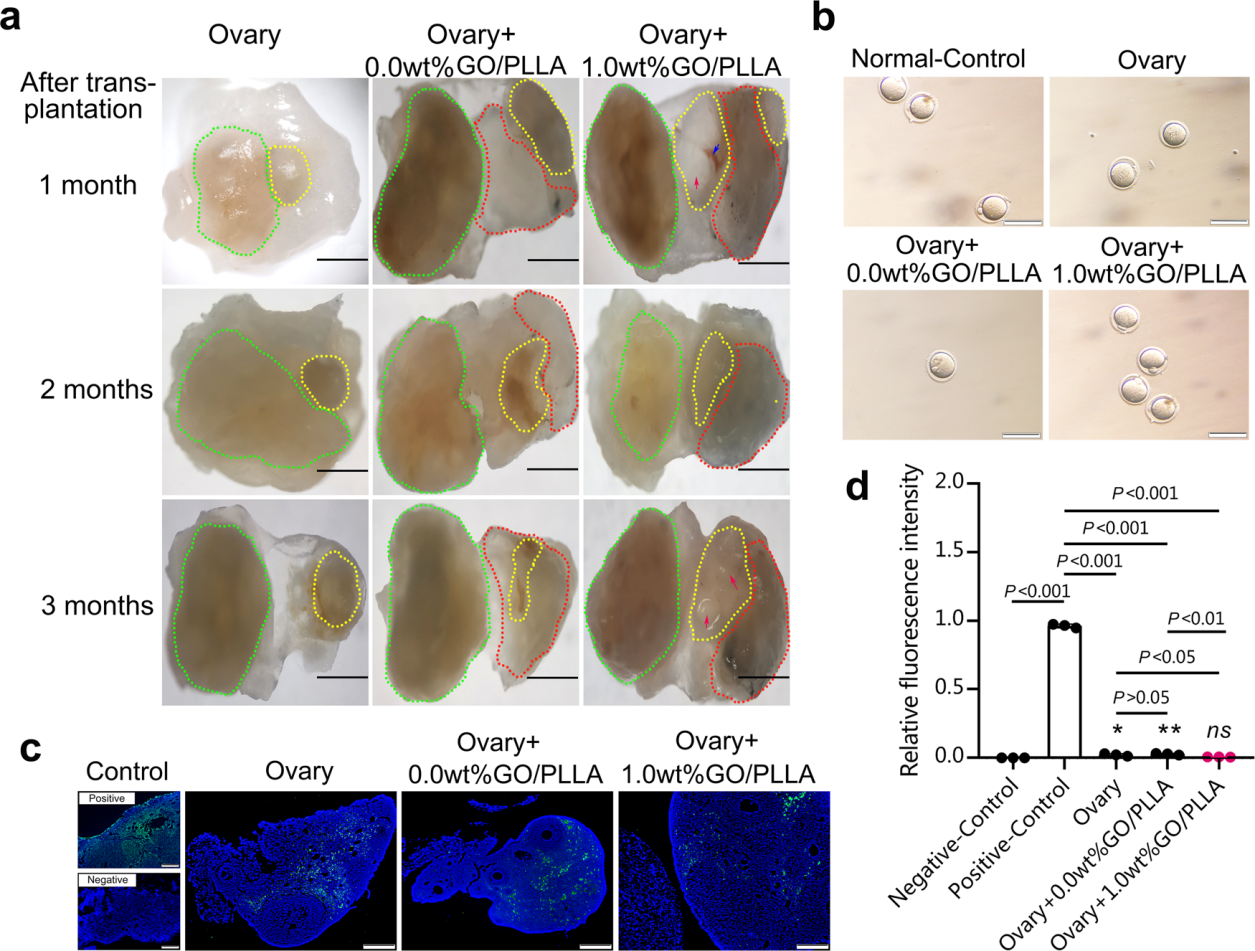
The in vivo survival of transplanted ovaries transplanted with GO/PLLA nanofiber scaffolds. **a** The green dotted line indicates the in situ ovarian tissue, the yellow dotted line indicates the implanted ovarian tissue, and the red dotted line indicates the material transplanted into the body. The blue arrow shows the blood vessel. Red arrow shows follicles, scale bar: 500 μm. **b** The most representative images of oocytes for IVM in the ovary transplantation group and co-transplantation groups. scale bar: 100 μm. **c** The TUNEL assay in each group after transplantation. scale bar: 200 μm. **d** The average IntDen of TUNEL-positive ovarian cells. Statistical significance levels are set at *P < 0.05, **P < 0.01 by one-way ANOVA (Multiple comparisons). Data are presented as mean ± SD, “ns” represent non-significant difference.

The POI mice were randomly divided into three groups: an ovary transplantation group (abbreviated as “Ovary”), a co-transplantation group of ovaries and 0.0 wt% GO/PLLA (Ovary+0.0 wt% GO/PLLA) and a co-transplantation group of ovaries and 1.0 wt% GO/PLLA (Ovary+1.0 wt% GO/PLLA). Normal ovaries loaded on GO/PLLA nanofiber scaffolds were transplanted into the ovarian cysts of POI mice with half of the ovary removed in advance, and the ovary transplantation group was regarded as the control group. At 1 month, 2 months and 3 months after ovarian transplantation, the survival rate of the transplanted ovarian tissue was observed by stereomicroscope. The results showed that prolongation of transplanted time could not promote the fusion of the pale yellow and hard transplanted ovaries with in situ ovarian tissue in the control group (Fig. 3a). However, in the co-transplantation group, the transplanted ovarian tissue could be fused with 0.0 wt% GO/PLLA and the in situ ovarian tissue (Fig. 3a). In addition to fusion, when the transplanted ovaries were loaded on 1.0 wt% GO/PLLA, obvious follicles could be seen on the surface of transplanted ovaries with distinct angiogenesis between the implanted ovarian tissue and the membranes, suggesting the survival of transplanted ovarian tissue (Fig. 3a). In addition, the ovary survival rate in the Ovary+1.0 wt% GO/PLLA group (*n* = 15, 86.7%) was significantly higher than those in the Ovary+0.0 wt% GO/PLLA (*n* = 13, 53.8%) and Ovary groups (*n* = 14, 28.6%, Supplementary Table 2).

To address the effect of GO/PLLA nanofiber scaffolds on oocytes, ovarian tissue on the treated side was further harvested from the three transplanted mice, and germinal vesicle (GV) stage oocytes were extracted and incubated with MII-stage oocytes in vitro. Thimbleful GV stage oocytes were found in the Ovary group compared with those in the co-transplantation groups, which were shown in Supplementary Table 3. After in vitro maturation (IVM) for 16 h, the extrusion of the first polar body (PB1) was obviously lower in the Ovary group (0.0%) and Ovary+0.0 wt% GO/PLLA group (50.0%) than that in the oocytes derived from the normal mice (referred to as the Normal-Control group, 83.3%), but without obvious change in the Ovary+1.0 wt% GO/PLLA group (85.7%) (Fig. 3b and Supplementary Table 3). In addition, this result confirmed that the average IntDen of TUNEL-positive ovarian cells in the 1.0 wt% GO/PLLA group was significantly weaker than those in the Ovary group and Ovary+0.0 wt% GO/PLLA group, and no statistically significant difference was proven in ovarian cell apoptosis compared with the Negative-Control group (Fig. 3c, d). All of the above results further suggested better survival of ovarian tissue transplanted with 1.0 wt% GO/PLLA nanofiber scaffold.

### Co-transplantation of ovarian tissue loaded on 1.0 wt% GO/PLLA nanofiber scaffold improves ovarian function and increases follicle number

**Fig. 4:**
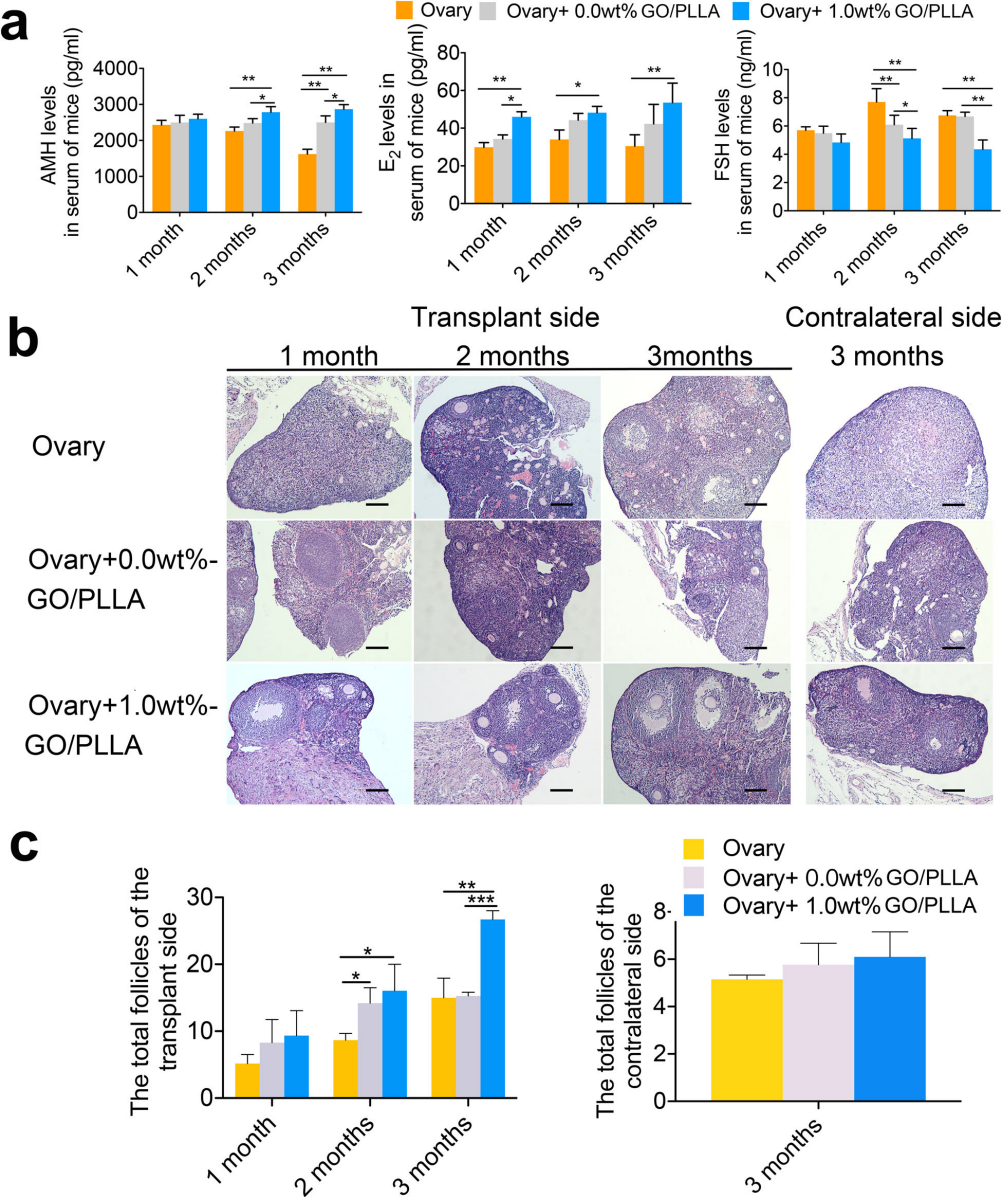
The hormone levels and total number of follicles at different time points after co-transplantation in the three groups. **a** Changes in serum hormone levels in POI mice after transplantation of normal ovarian tissue. **b** Representative images of H&E staining of ovarian tissue in each group at 1, 2 and 3 months after transplantation. **c** The number of follicles in the transplanted and contralateral ovarian tissues. Statistical significance levels are set at *P < 0.05, **P < 0.01, ***P < 0.001 by one or two*-*way ANOVA (Multiple comparisons). Data are presented as mean ± SD, scale bar: 200 μm.

To further confirm the noticeable impact of implanted ovarian tissue on ovarian function in POI mice, ELISA detection was used to determine the serum hormone levels of POI mice at 1, 2, and 3 months after transplantation. The results showed that E_2_ (estradiol) levels in the Ovary+1.0 wt% GO/PLLA group (28.50 ± 5.07 pg/mL) were dramatically higher than those in the Ovary group (18.76 ± 2.14 pg/mL) and Ovary+0.0 wt% GO/PLLA group (19.38 ± 3.44 pg/mL) at 1 month after transplantation, but there was no significant difference in FSH (follicle-stimulating hormone) and AMH (anti-miillerian hormone) levels among the three groups. After transplantation for 2 months, E_2_ levels increased significantly in the Ovary+1.0 wt% GO/PLLA group (41.27 ± 3.32 pg/mL) compared with those in the Ovary group (20.18 ± 2.18 pg/mL). In particular, higher AMH levels and significantly reduced FSH levels were found compared with the Ovary and Ovary+0.0 wt% GO/PLLA groups. As the implantation time increased to 3 months, compared with the Ovary group (20.76 ± 3.70 pg/mL), E_2_ still maintained a significantly increased level in the Ovary+1.0 wt% GO/PLLA group (46.44 ± 5.28 pg/mL), as did AMH levels (2.30 ± 0.38 ng/mL) compared to both the Ovary group (0.82 ± 0.17 ng/mL) and Ovary+0.0 wt% GO/PLLA group (0.98 ± 0.23 ng/mL). In addition, FSH levels (21.50 ± 2.05 ng/mL) in the Ovary+1.0 wt% GO/PLLA group were lower than those in the Ovary group (41.84 ± 3.54 ng/mL) and Ovary+0.0 wt% GO/PLLA group (37.15 ± 2.46 ng/mL). These results indicated that the co-transplantation of ovarian tissue and GO/PLLA nanofiber scaffolds could recover ovarian function, and a conspicuous effect was verified in the Ovary+1.0 wt% GO/PLLA group (Fig. 4a).

The in vivo survival of the implanted ovarian tissue (transplant side) and the development of follicles in situ ovarian tissue (contralateral side) were observed. Follicle counting showed that no obvious difference was found in the total follicles on the transplant side between the three groups after transplantation for 1 month; however, they were significantly increased in the Ovary+1.0 wt% GO/PLLA group and Ovary+0.0 wt% GO/PLLA group compared with the Ovary group, and no significant difference was found between the Ovary+1.0 wt% GO/PLLA group and the Ovary+0.0 wt% GO/PLLA group. At 3 months after transplantation, the total number of follicles in the Ovary+1.0 wt% GO/PLLA group was significantly higher than those in the Ovary+0.0 wt% GO/PLLA group and Ovary group (Fig. 4b). In addition, a small number of follicles were found after transplantation for 3 months in the contralateral ovarian tissue of the three groups, without a significant difference between the three groups (Fig. 4c).

### Co-transplantation of ovarian tissue loaded on 1.0 wt% GO/PLLA nanofiber scaffold promotes ovarian angiogenesis and characteristic changes of GO/PLLA nanofiber scaffold in vivo

**Fig. 5:**
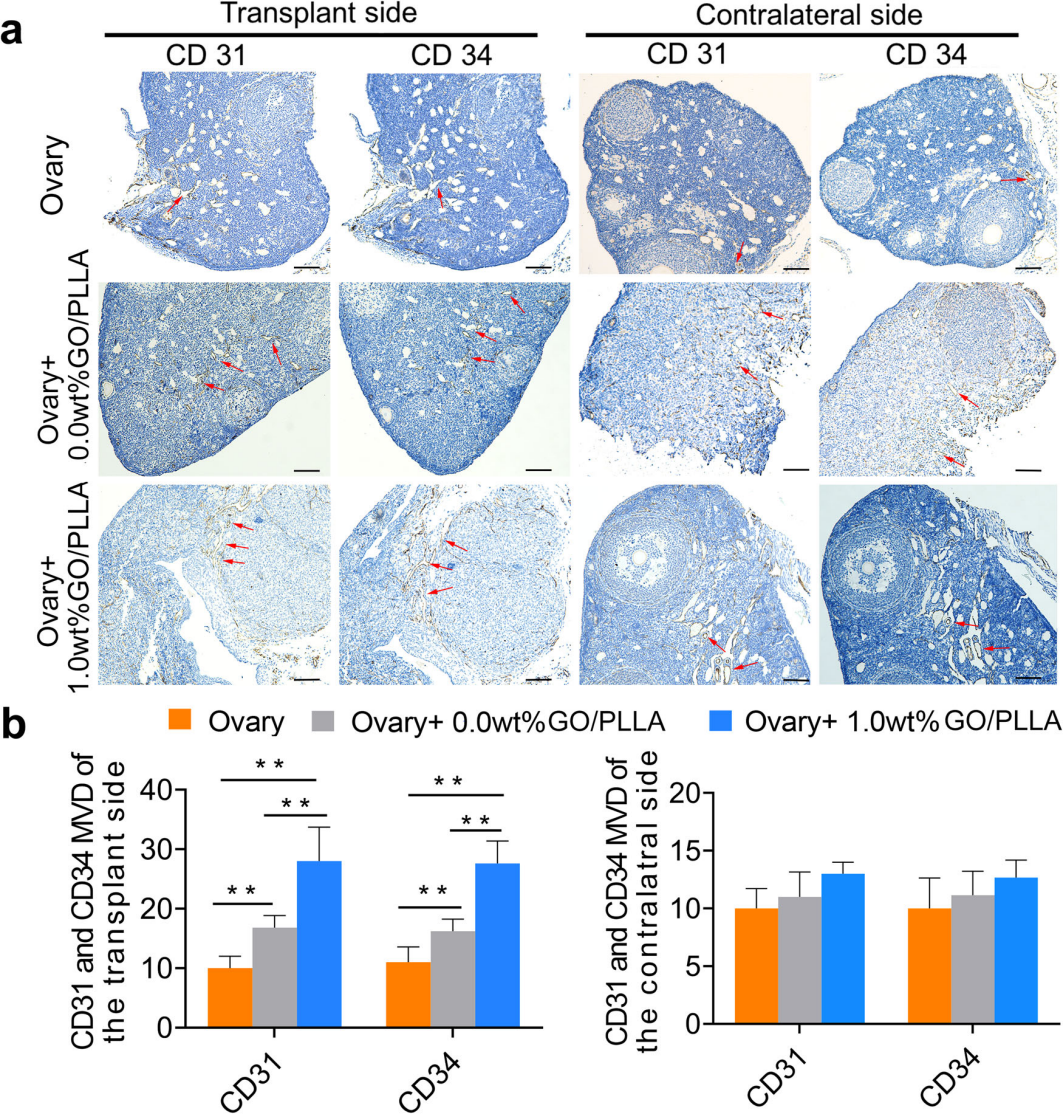
The expression and MVD (per 200X field) of CD31 and CD34 in the transplant side and contralateral ovaries in the three groups. **a** Localization and expression of CD31 and CD34 in ovarian tissue of the three groups after transplantation for 3 months. Red arrows indicate blood vessels. **b** Comparison of MVD by CD31 and CD34 between the three groups. Statistical significance levels are set at **P < 0.01 by one-way ANOVA (Multiple comparisons). Data are presented as mean ± SD, scale bar: 200 μm.

Immunohistochemistry assays were used to detect the expression levels of vascular-specific molecules related to angiogenesis (such as CD31 and CD34) in ovarian tissue 3 months after transplantation (Fig. 5a). The microvessel density (MVD, per 200X field) showed that both CD31 and CD34 expressions in the Ovary+1.0 wt% GO/PLLA group were conspicuously higher than those in the Ovary+0.0 wt% GO/PLLA group and Ovary group; moreover, increasing expression levels were found in the Ovary+0.0 wt% GO/PLLA group compared with those in the Ovary group (Fig. 5b). However, there was no significant difference in CD31 and CD34 expression in the contralateral ovarian tissue among the three groups (Fig. 5b).

**Fig. 6:**
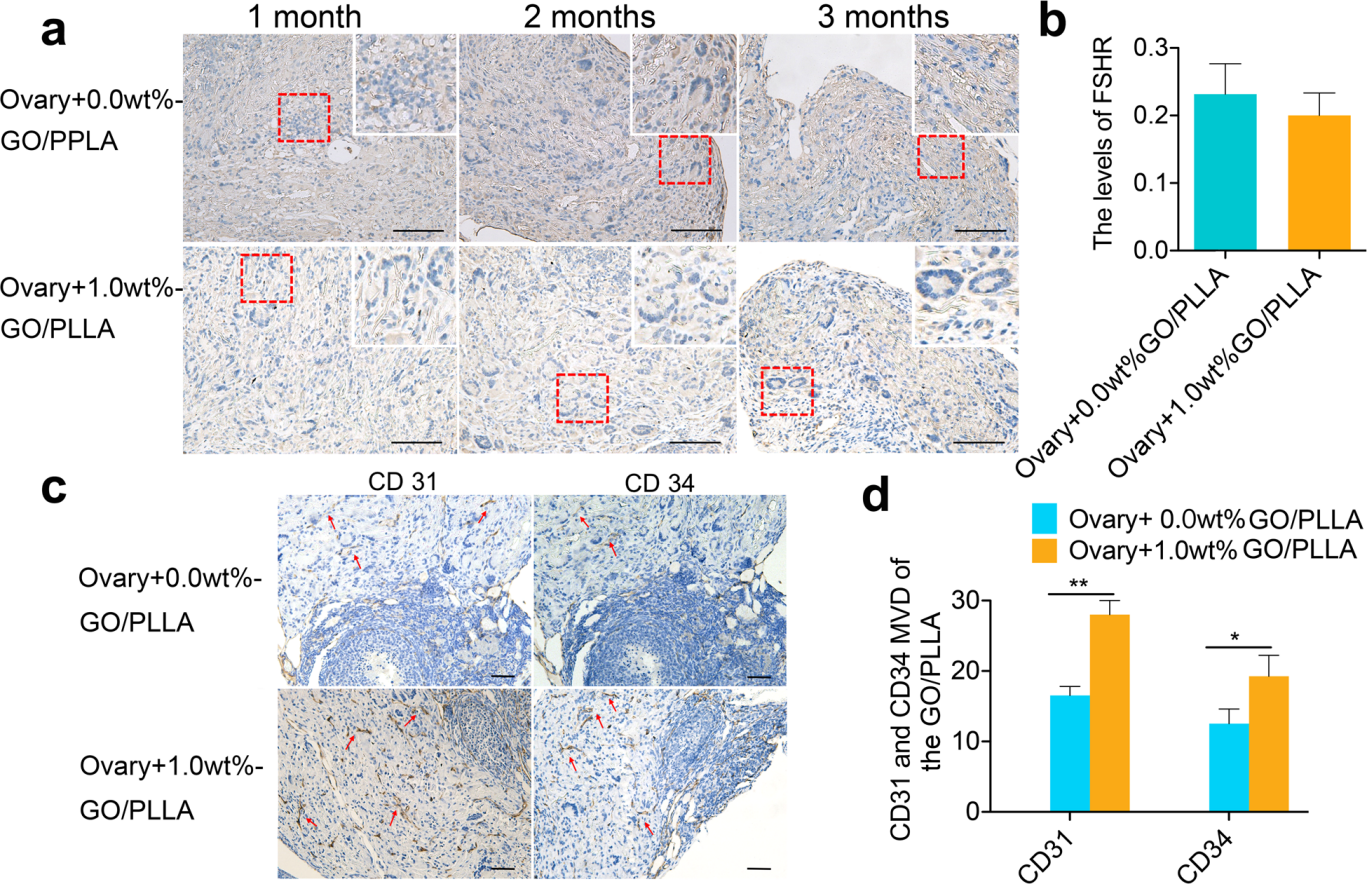
FSHR expression and MVD (per 200X field) changes on the GO/PLLA nanofiber scaffolds. **a** Representative images of immunohistochemical staining for FSHR show the growth of GCs on the 0.0 wt% and 1.0 wt% GO/PLLA nanofiber scaffolds. The dotted red frame shows the growth of GCs in a circular pattern. **b** The expression levels of FSHR on 0.0 wt% and 1.0 wt% GO/PLLA nanofiber scaffolds. **c** Localization and expression of CD31 and CD34 on the 0.0 wt% and 1.0 wt% GO/PLLA nanofiber scaffolds. The red arrows indicate blood vessels. **d** Comparison of MVD by CD31 and CD34 on 0.0 wt% and 1.0 wt% GO/PLLA nanofiber scaffolds. Statistical significance levels are set at *P < 0.05, **P < 0.01 by two-tailed Student’s t-test, Data are presented as mean ± SD, scale bar: 200 μm.

To reveal the possible role of GO/PLLA nanofiber scaffolds in promoting the survival of transplanted ovarian tissue, we specifically characterized the changes in the scaffolds in vivo. In this study, it was found that some follicle-stimulating hormone receptor (FSHR)-positive cells were scattered across the membrane in the Ovary+1.0 wt% GO/PLLA and Ovary+0.0 wt% GO/PLLA groups, but there was no significant difference between the two groups, which at least suggested the presence of GCs on the GO/PLLA nanofiber scaffolds (Fig. 6a, b), also indicating good biocompatibility in vivo of GO/PLLA nanofiber scaffolds. In addition, the immunohistochemical analysis further showed the expression of CD31 and CD34 on the nanofiber scaffolds with higher expression levels in Ovary+1.0 wt% GO/PLLA group than that in the Ovary+0.0 wt% GO/PLLA group (Fig. 6c, d), further suggesting that these nanofiber scaffolds displayed the changes in angiogenesis after in vivo transplantation, especially for the Ovary+1.0 wt% GO/PLLA group.

### GO/PLLA nanofiber scaffolds accelerate NO production by p-eNOS in vivo

**Fig. 7:**
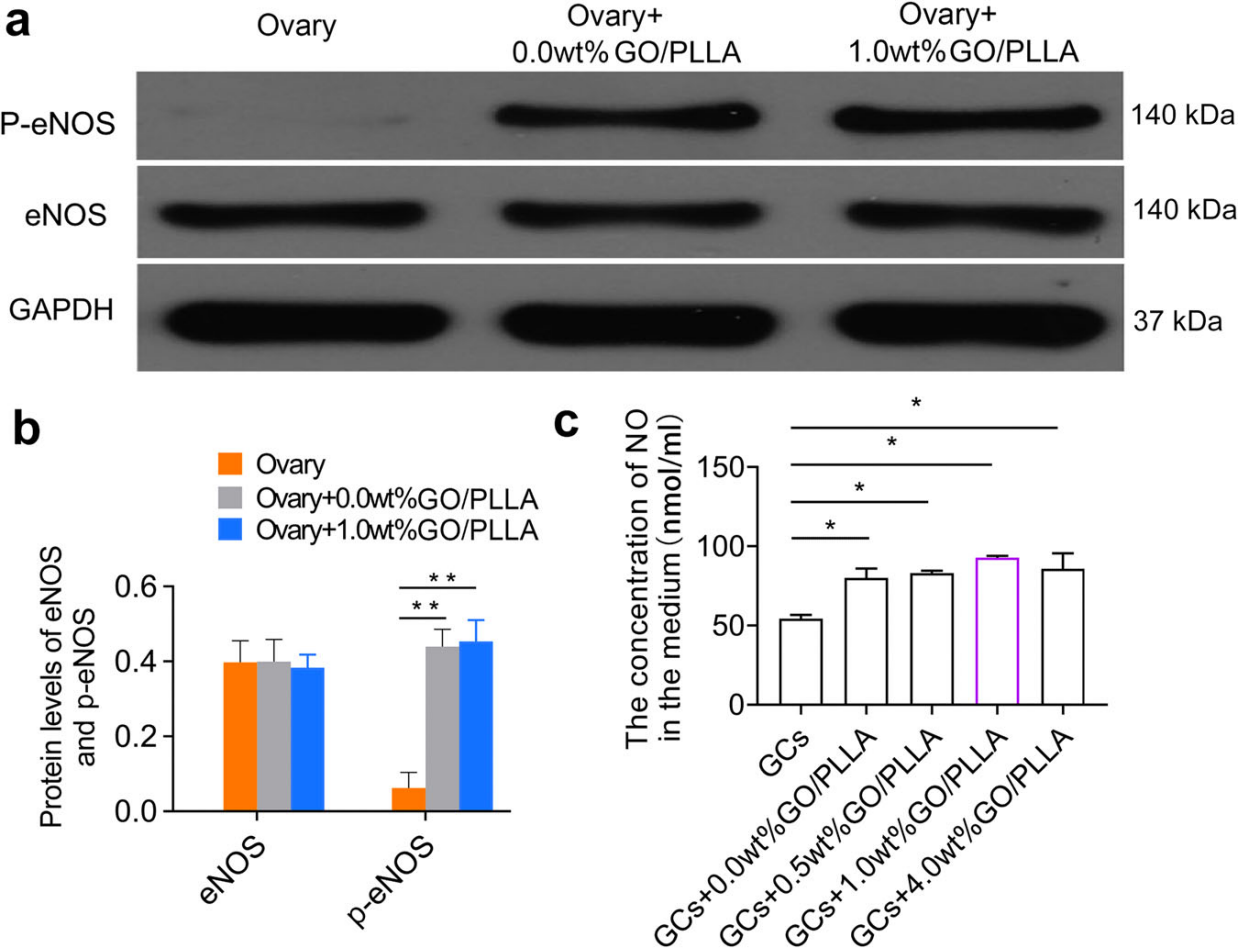
eNOS and p-eNOS expression levels in transplanted ovarian tissue in the three groups and NO production in the supernatant of granulosa cells co-cultured with different concentrations of GO/PLLA nanofiber scaffolds. **a** Representative image of western blot. **b** Protein levels of eNOS and p-eNOS based on the relative gray value of the western blot. **c** The concentration of NO in the supernatant of GCs co-cultured with different concentrations of GO/PLLA nanofiber scaffolds. All the western blots were confirmed to be derived from the same experiment and processed in parallel. Statistical significance levels are set at *P < 0.05, **P < 0.01 by one-way ANOVA (Multiple comparisons). Data are presented as mean ± SD.

Given the marked effect of GO/PLLA nanofiber scaffolds on angiogenesis, which played a potential and fundamental role in the survival and function of transplanted ovarian tissue, we further investigated the molecular pathway of GO/PLLA nanofiber scaffolds in promoting angiogenesis. Western blotting was performed to detect eNOS and p-eNOS expression levels in ovarian tissue (transplanted side) of the three groups (Fig. 7a), showing that protein expression levels of p-eNOS obviously increased in the Ovary+1.0 wt% GO/PLLA and Ovary+0.0 wt% GO/PLLA groups compared to the Ovary group (Fig. 7b). However, no significant difference was found in the eNOS expression levels among the three groups (Fig. 7b). Furthermore, NO levels were detected by an NO detection kit in GCs culture supernatant, in which GCs were co-cultured with multigradient concentrations of GO/PLLA nanofiber scaffolds. Compared with the control group (GCs), a significantly higher NO concentration was found in the 0.0 wt%, 0.5 wt%, 1.0 wt% and 4.0 wt% GO/PLLA nanofiber scaffolds with the most obvious upward trend in the GCs+1.0 wt% GO/PLLA group (Fig. 7c), but no difference was showed between the GCs+1.0 wt% GO/PLLA group and the GCs+ 0.0 wt% GO/PLLA group, which was consistent with the above result of p-eNOS expression levels.

## Discussion

Due to the unique physicochemical and mechanical properties of GO, as well as their good biocompatibility, a series of novel GO/PLLA nanofiber scaffolds with high performance was prepared by mixing GO with PLLA followed by an electrospinning process (Supplementary Figs. 4, 5). GO, as one form of graphene derivative, can be regarded as an amphiphilic two-dimensional material with an edge-to-center distribution of hydrophobic and hydrophilic domains^32,33^. Given that PLLA has nonpolar and hydrophobic groups, attractive hydrophobic interactions between GO and PLLA occur at the hydrophobic domain of GO nanosheets, thus significantly enhancing its mechanical properties (Fig. 1a and Supplementary Fig. 7). Moreover, these hydrophilic oxygen functional groups could improve the dispersibility of GO/PLLA nanofiber scaffolds, thus avoiding remarkable sedimentation or phase separation during the mixing process. This enabled the formation of continuous, uniform, and defect-free nanofibers with smooth surfaces, and no GO nanosheets protruded from the nanofiber surface (Fig. 1b, c and Supplementary Fig. 8). The results suggested that the presence of strong interfacial interactions in the composites conferred GO/PLLA nanofiber scaffolds with enhance mechanical properties. This was further demonstrated by Raman spectroscopy analysis (Supplementary Fig. 6). When the mass ratio of GO was 1.0 wt%, Young’s modulus and strength of the nanofiber scaffolds increased to 1.09 GPa and 1.67 MPa, which was higher than those of pure PLLA. However, at the 4.0 wt% GO content level, Young’s modulus slightly decreases, which may be attributed to the aggregation of GO nanosheets that caused the reduced crystallinity of PLLA and thus limited the interfacial load transfer (Supplementary Fig. 7a). This was in good agreement with the trend of the stress-strain curves (Supplementary Fig. 7b). Furthermore, the water contact angles of the nanofiber scaffolds changed from 66.7° to 55.6° with increasing GO concentration, suggesting that incorporation of GO into PLLA matrix made the surface more hydrophilic (Fig. 1d and Supplementary Fig. 9a). The porosity of these nanofiber scaffolds was relatively high, ranging from 0.81 to 0.84. (Supplementary Fig. 9b) The hydrophilic surface and the porous network structure of GO/PLLA nanofiber scaffolds made it possible to be highly favorable towards cell infiltration and after implantation. Together, these results suggested that, when the mass ratio of GO was less than 1.0 wt%, nanosheets were well dispersive in PLLA matrix, leading not only to improve the mechanical performance of PLLA but also to increase its hydrophobicity, consequently facilitating the increase of the proliferation and the promotion of the differentiation and angiogenic potential of PLLA for ovarian transplantation.

We proved that the survival rate of transplanted tissue could be improved after the ovarian cortex was implanted in situ combined with the new GO/PLLA nanofiber scaffolds in POI mice. Moreover, the establishment of blood supply saved the loss of large numbers of follicles in the transplanted ovarian tissue despite imperfect end-to-end anastomosis. Previous studies have reported that some antioxidants (such as vitamin C, etc.), angiogenesis factors, and ExECs have been proven to have transitory effects on the survival of grafts^16,20,34–37^. In this study, ovarian tissue co-transplanted with 0.0 wt% GO/PLLA nanofiber scaffold was fused with in situ ovarian tissue with pink tissue and soft texture. Obviously, protrusive follicles and vascular structures were found on the surface of transplanted ovarian tissue after co-transplantation with 1.0 wt% GO/PLLA nanofiber scaffold. However, the ovarian cortex transplanted alone existed in isolation without any fusion at 1 to 3 months after transplantation in situ, as well as being yellowish in the color of the transplanted ovarian tissue, even hard in texture. Moreover, the in vitro developmental potential of oocytes inspired by the transplanted ovaries loaded on the GO/PLLA nanofiber scaffolds was improved compared with that in the ovary transplantation group, especially for the inhibited tissue apoptosis after transplantation combined with the new composite materials. The results suggested that the ovarian cortex was more likely to survive than the ovarian tissue transplanted alone in the Ovary+0.0 wt% GO/PLLA or Ovary+1.0 wt% GO/PLLA groups, which preliminarily indicated that GO/PLLA nanofiber scaffolds had a positive effect on the transplanted ovarian tissue, conducive to organ transplantation. To further confirm the restoration of ovarian dysfunction caused by chemotherapy drugs, steroid hormone levels were detected to show the initial obvious difference (increased AMH levels and declined FSH levels) at two months after co-transplantation with 0.0 wt% GO/PLLA and 1.0 wt% GO/PLLA nanofiber scaffolds, with an especially significant improvement in the Ovary+1.0 wt% GO/PLLA group compared with the Ovary+0.0 wt% GO/PLLA group, indicating that both GO and PLLA had curative effects on promoting the survival of transplanted ovarian tissue and that the combined application could have a better effect on the restoration of ovarian function. In addition, the total follicles were also counted in the transplanted ovary, and the results were consistent with the hormone levels. Although there was no difference in the number of follicles among the three groups after transplantation for one month, the total number of follicles began to increase at the second month in the Ovary+0.0 wt% GO/PLLA and Ovary+1.0 wt% GO/PLLA groups and did not show a marked increase compared with that in the Ovary group until the third month, especially in the Ovary+1.0 wt% GO/PLLA group, indicating that the addition of GO was more conducive to follicular development and growth. The same results were also observed in contralateral ovarian tissue. A small number of follicles were discovered in the impaired ovaries from the separate transplantation group, but it showed an increasing trend compared with the separate transplantation group in the 1.0 wt% GO/PLLA co-transplantation group, suggesting that its functional recovery was due to the regulation of the HPO axis, which was affected by ovarian function on the transplant side.

Scaffold materials are an important part of research in the field of tissue engineering. They can contribute to cell survival in vitro/in vivo by providing a 3D growth environment and mechanical support and are extremely important for cell adhesion, growth, proliferation, differentiation, and other life activities^38,39^. We further revealed the mechanism by which GO/PLLA nanofiber scaffolds could promote the survival of transplanted ovarian tissue. CD31 and CD34 are typical vascular-specific molecules, the expression levels of which could indicate the formation of blood vessels^40,41^. The results showed that CD31 and CD34 in ovarian tissue of the transplant side were highly expressed both in the Ovary+1.0 wt% GO/PLLA and Ovary+0.0 wt% GO/PLLA groups, which was significantly higher than that of the separate transplantation group, indicating that both nanofiber scaffold promoted angiogenesis and 3D environment may be conducive to the establishment of microvessels. It is now widely accepted that the establishment of collateral vessel networks can improve local blood perfusion and play a key role in the survival of tissue transplantation^14^. It was reported in diabetic patients that the hindlimb microvasculature could be established by activating the eNOS/NO pathway; p-eNOS promoted the production of NO and thus regulated angiogenesis^42–44^. It has been proven that low-dose graphene can produce trace amounts of NO by promoting the formation of eNOS^21^. Therefore, we detected the levels of NO in the supernatant of granulosa cells co-cultured with nanofiber scaffolds. It was found that, compared to the GCs group, nanofiber scaffolds could contribute to the secretion of NO, with the most obvious elevated NO levels in the GCs+1.0 wt% GO/PLLA group. Simultaneously, compared with the ovary group, the expression levels of p-eNOS in the transplanted ovarian tissue were also detected and revealed a higher expression in the Ovary+1.0 wt% GO/PLLA group and Ovary+0.0 wt% GO/PLLA group. However, only a slight upward trend of p-eNOS expression was showed in the Ovary+1.0 wt% GO/PLLA group compared with Ovary+0.0 wt% GO/PLLA group, in accordance with NO levels in the supernatant of co-cultured cells, indicating that p-eNOS may be involved in the regulation of NO production and play a certain role in promoting angiogenesis related to GO/PLLA nanofiber scaffolds. Moreover, it was also suggested that eNOS/NO signaling pathway might be one of the causes of the pro-angiogenesis effect. According to the current studies, it was believed that the angiogenic effect of GO was mostly confined to its delivery of relative growth factors, such as CD31, CD34, VEGF, MMP9, and SDF-1, and it had been also shown that GO could activate endothelial tip cells and promote angiogenesis by binding endogenous lysophosphatidic acid^45^, which may be another reason for better pro-angiogenesis effect in the 1.0 wt% GO/PLLA group compared with 0.0 wt% GO/PLLA group.

PLLA, as the most widely used degradable polymer, can be finally converted into carbon dioxide and water, thus having no harmful impact on the environment^46–48^. The biodegradation behavior of GO/PLLA nanofiber scaffolds was initially evaluated under aqueous and physiological environments. These results suggested that GO/PLLA nanofiber scaffolds had a negligible degradation rate in both H_2_O and PBS (Supplementary Figs. 10–13). After 28 days, no significant changes in the morphology or microstructure were observed, demonstrating that they were very stable in H_2_O and PBS. However, when immersed in DMEM and DMEM/FBS, their surface became rough over a period of 14 days, and the well-formed and dense PLLA gradually swelled, subsequently undergoing deformation and partial deterioration. More importantly, the presence of GO nanosheets could significantly retard the decomposition of GO/PLLA nanofiber scaffolds under the same conditions, and the variation in swelling was not observed until 21 days after immersion. This was likely because it took more time for ions as well as proteins to diffuse and then permeate into the surface of nanofibers and the phase interfaces. In the case of 4.0 wt% GO/PLLA nanofiber scaffold, no variations in the morphology and structures were observed. Therefore, GO nanosheets were able to improve the barrier properties of PLLA matrix, in turn enabling control of the degradation rate. Furthermore, when GO/PLLA nanofiber scaffolds were transplanted into the body, SEM images showed that 0.0 wt% GO/PLLA nanofiber scaffold underwent swelling and began to form merged areas, and their diameter gradually increased over time, showing remarkable degradation in vivo largely due to the penetration of water, ions, and proteins into PLLA matrix (Supplementary Fig. 19). For 1.0 wt% GO/PLLA nanofiber scaffold, however, there were no significant differences in morphology and size. This suggested that the presence of GO nanosheets makes the diffusion path more tortuous for water, ions, and proteins to reach the surface matrix, thus showing an obvious delaying effect on the degradation of PLLA^49,50^. Therefore, 1.0 wt% GO/PLLA nanofiber scaffold showed higher biopersistence and was suitable for use as a tissue scaffold.

In addition, granulosa cells could migrate to the nanofiber scaffolds and were distributed in a circle, similar to the distribution characteristics of granulosa cells around the oocyte in the ovary, because polylactic acid material could make granulosa cells better maintain their cell characteristics, suggesting good histocompatibility, but the specific mechanism remained unknown and needs further study. Moreover, a certain amount of angiogenesis was found on GO/PLLA nanofiber scaffolds, especially in the Ovary+1.0 wt% GO/PLLA group, further corroborating that GO in the nanocomposites could quickly establish the microvascular network of ovarian tissue, which could strengthen ovarian tissue implantation and accelerate blood perfusion. This may be one of the reasons why the transplanted tissues were more likely to survive and had the best ovarian function in the Ovary+1.0 wt% GO/PLLA group.

In this study, we established a new type of GO/PLLA nanofiber scaffolds that could be applied to the technique of ovarian cortex transplantation and accelerate the construction of a microvascular network in transplanted tissue with a certain proportion of graphene so that the ovarian tissue was implanted faster to restore ovarian function and restore the function of the contralateral ovary to a certain extent. In addition, the nanofiber scaffolds could be degraded to some extent in vivo and gradually became integrated with the ovarian tissue, and biological behaviors such as cell migration, growth and blood vessel establishment occurred, indicating that the material had good biocompatibility. The establishment of these nanofiber scaffolds not only provided a new strategy for female fertility preservation but also provided a new method as a vector for organ transplantation.

## Methods

### Materials

Nature graphite (99%), dichloromethane (DCM), and N,N-dimethylformamide (DMF) were purchased from Alfa Aesar. Poly-L-lactic acid (PLLA, fiber-grade, inherent viscosity (*η*_inh_) = 2.2 dl g^−1^, measured at 0.1% w/v in chloroform (CHCl_3_) at 25 °C, Mn = 300,000) was supplied by Jinan Daigang Biomaterial Co., Ltd. (Shandong, China). Other chemicals were obtained from Beijing Chemical Reagent Co. All reagents were of analytical grade and were applied as received without further purification. Deionized (DI) water (resistivity (*ρ*) ≥ 18.2 MΩ cm at 25 °C) was supplied by the recirculating deionized water system (Arium® pro DI, Sartorius) for the experimental procedures.

### Animals

The approval was obtained from the Animal Research Center of Huazhong University of Science and Technology, and the study was carried out in accordance with the National Research Council’s “Guideline for the Care and Use of Laboratory Animals”. Female C57BL/6 mice (8 weeks old, 19–21 g) were purchased from the Experimental Animal Center of Three Gorges University (China). All mice were adapted for 3 days after their purchase and were maintained under a controlled temperature (26 ± 2 °C) with 12 h light/dark conditions and a standard pellet diet.

### Preparation of graphene oxide nanosheets

Graphene oxide (GO) was synthesized from natural graphite via a modified Hummer’s method followed by ultrasonication treatment^32,51^. In detail, graphite powder (0.5000 g) was added slowly into concentrated sulfuric acid (H_2_SO_4_, 50 mL, 98%) under ice-bath conditions. After 2 h of vigorous stirring in an ice bath, potassium permanganate (KMnO_4_, 1.5000 g) was gradually added to obtain a green mixture owing to the formation of the oxidizing agent MnO_3_^+^. Sequentially, the above mixture was kept at 37 °C for 2 h under strong stirring, accompanied by the disappearance of the green color. Hydrogen peroxide (H_2_O_2_, 10 mL, 30%) was then dropped dropwise into the above mixture to eliminate excess KMnO_4_. When stirred for 30 min, the resulting yellow dispersion was centrifuged at 12,000 × *g* for 30 min, and the precipitate was washed several times with 5% hydrochloric acid aqueous solution until almost no SO_4_^2−^ ions were detected by Ba^2+^ ions. The black precipitate was redispersed in DI water and placed into a dialysis bag (cut-off 3 000 Da) to dialyze 6 times a week for 2–36 h, completely removing any residual metal ions and acids. Finally, GO dispersion was obtained through bath sonication of the precursor aqueous solution of the as-made graphite oxide for 1 h, followed by centrifugation for 30 min at 12 000 × *g* to remove any unoxidized graphite flake or unexfoliated graphite oxide. The obtained supernatant was collected and subsequently freeze-dried for 2 days.

### Preparation of spinning dispersion of GO/PLLA nanocomposites

Initially, PLLA was completely dissolved in a binary-solvent system of DMF and DCM (1:1, volumetric ratio) and then stirred at room temperature (RT) for 12 h to achieve a uniform and transparent polymer solution (10 wt%). Meanwhile, GO (10 mg/mL) was dispersed into the same binary-solvent system with the help of ultrasonication for 2 h below 5 °C. Subsequently, an adequate volume of GO suspension was added slowly to the previously prepared PLLA solution and stirred at RT for another 12 h to achieve spinning dispersions of GO/PLLA nanocomposites with different concentrations of GO, including 0.5, 1.0, and 4.0 wt% with respect to the mass of PLLA. Prior to use, all the dispersions were homogenized in a bath sonicator for 0.5 h below 5 °C and then left to stand overnight.

### Electrospinning of GO/PLLA dispersions for nanofiber scaffolds

GO/PLLA nanofiber scaffolds were prepared via an electrospinning technique (HD-1311, Beijing Ucalery Technology Development Co., Ltd, China). The dispersions of GO/PLLA nanofiber scaffolds were placed in a commercial glass syringe (10 mL) fitted with a steel needle (27 G). Electrospinning was performed with a voltage of 15 kV supplied by a high voltage power, and a syringe pump was employed to feed the dispersion of GO/PLLA nanofiber scaffolds into the needle tip at a speed of 0.30 mm min^−1^. The electrospun nanofibers were collected on a target drum placed 20 cm apart from the syringe tip, and the roller rotation speed was 120 × *g*. All experiments were conducted at 25 °C and with a relative humidity of ~50%. Finally, the samples were freeze-dried for 48 h to remove any organic chemicals and then cut into the same specification standby.

### Physical characterization

Electrospun GO/PLLA nanofiber scaffolds were quickly coated with a thin palladium layer using an ion sputter (E-1045, Hitachi High-technologies, Japan), and their morphology was analyzed by field-emission scanning electron microscopy (FE-SEM, S-4800, Hitachi High-technologies, Japan) with an accelerating voltage of 1 kV. AFM images were collected with a Bruker Multimode 8 AFM (Bruker, USA) in ScanAnalyst mode (spring constant 0.35 N m^−^^1^ and frequency 65 kHz). XPS measurements were conducted with an ESCALab220i-XL spectrometer equipped with a twin-anode Al-K_α_ X-ray source (1486.6 eV). Micro-Raman spectra were obtained from Raman Spectroscope (Renishaw inVia plus, UK) with excitation at 514 nm. The contact angles were measured at RT in ambient air using an automatic contact angle goniometer coupled with a flash camera (DSA 100, Krüss, Germany, DI water as test liquid). Porosity was determined using the segmental method^46^. Mechanical testing of nanofiber scaffolds was performed on the universal testing machine Zwick 1435 (Zwick Roell, Germany).

### In vitro cytotoxicity study

The cytotoxicity of GO nanosheets was assessed in granulosa cells using a Cell Counting Kit-8 (CCK-8) assay. First, granulosa cells (5 × 10^3^ cells per well) were seeded in Dulbecco’s modified Eagle’s medium (DMEM, HyClone, USA) supplemented with 10% fetal bovine serum (FBS, Gibco, Shanghai, China) and 1% penicillin-streptomycin solution (100 units/mL penicillin and 100 mg/mL streptomycin, GE Life Science) at 37 °C under a humidified atmosphere with 5% CO_2_. After cultivation for 12 h, these cells were treated with GO nanosheets at various concentrations (0, 2.5, 5, 10, 20, 30, 40, 50, 75, and 100 μg/mL) for another 24 h. These treated cells were washed three times with PBS and incubated with CCK-8 reagent (10%, Beyotime, China) in serum-free medium for several hours. Finally, the optical density of each well was measured by a microplate spectrophotometer (SpectraMax M2MDC, USA) at 450 nm.

Furthermore, the cytotoxic effect of various concentrations of GO nanosheets was evaluated in granulosa cells using Live-Dead staining based on calcein AM/propidium iodide (CA/PI). Granulosa cells were seeded in 24-well plates at a density of 3 × 10^4^ cells per well for 12 h and treated with GO nanosheets at final concentrations of 0, 5, 10, 20, 50, and 100 μg/mL. After incubation for another 24 h, these treated cells were stained with CA/PI for 20 min, and an inverted fluorescence microscope (Olympus X73, Tokyo, Japan) was applied to collect fluorescent images.

### In vitro degradation assay

To simulate the degradation behavior of nanofiber scaffolds, all samples, including pure PLLA nanofiber scaffold and GO/PLLA nanofiber scaffolds with different GO concentrations, were first cut into square pieces (10 mm × 10 mm) and then immersed in bottles containing 5 mL of DI water, phosphate buffer solution (PBS, pH 7.40), DMEM, or DMEM supplemented with 10% FBS. Subsequently, these bottles were placed into a rotary shaker and shaken at 37 °C (200 × *g*). At selected time intervals (3, 7, 14, 21, and 28 days), samples were then removed, gently rinsed several times with DI water, and dried at RT for one week. Finally, dried nanofiber scaffolds were analyzed using SEM and Raman spectroscopy.

### Isolation and culture of granulosa cells

Granulosa cells (GCs) were extracted from the ovaries of female C57BL/6 mice at 8 weeks old. The ovaries were removed from the mice and placed in M2 medium (Sigma, USA). Isolated ovaries were washed twice under a stereomicroscope (XTL-400, China). GCs were collected by puncturing follicles from the isolated ovary and cultured in DMEM supplemented with 10% FBS and 1% penicillin and streptomycin in a humidified cell culture incubator (37 °C, 5% CO_2_). GCs were identified by the specific molecular FSHR (1:500; Proteintech, Cat No. 22665-1-AP, China) and seeded on cell culture plates and GO/PLLA nanofiber scaffolds, and the growth status was observed after adherence.

### POI model construction

Cisplatin (Sigma, USA) was used to establish the POI model. Wild-type C57BL/6 female mice (8 weeks) were randomly divided into two groups: the control group (*n* = 10) and the POI group (*n* = 90). The mice divided into the POI group (cisplatin treatment) received daily intraperitoneal (i.p.) injections of cisplatin (1.5 mg/kg) for 10 days, and equivalent doses of normal saline were injected as the control. The weight of the mice was recorded before and after the experiment, and the blood and ovaries were collected to test the hormone levels to analyze the total follicles. Serum extracted from the two groups was stored at −80 °C to be used for enzyme-linked immunosorbent assay (ELISA). The ovarian tissues were immediately preserved in liquid nitrogen and fixed with a 4% paraformaldehyde solution.

### Ovarian tissue transplantation

After a unilateral half of the ovarian tissue was removed from the recipient POI mice, another unilateral half of the normal ovarian tissue from the donor normal mice was obtained and cut into equal-sized blocks, and then transplanted in situ into the ovarian cysts of POI mice at the removed side. Specifically, the POI mice were anesthetized by intraperitoneal injection of 1% pentobarbital sodium (50 mg/kg) under aseptic conditions, followed by routine disinfection. The skin and muscle were incised and opened at the coastal ridge angle to fully expose the right fallopian tube and ovarian tissue. Then, a small incision was made in the ovarian surface envelope (referred to as “cyst”) with microsurgical scissors, and ovarian tissue blocks from normal mice or the blocks loaded on the GO/PLLA nanofiber scaffolds were implanted into the cyst with microsurgical tweezers. The ovarian cyst was kneaded with hot microsurgical tweezers, and the back incision was sutured. Then, ovarian tissue was obtained 1, 2 and 3 months after transplantation.

### Oocytes collection and maturation

Following 10 IU pregnant mare serum gonadotrophin (PMSG, Bohn Pharmaceutical Co., Ltd., China) via intraperitoneal injection for 46–48 h, the GV stage oocytes were collected by puncturing the ovarian follicles with freshly isolated cumulus-Oocyte complexes (COCs) and cultured in equilibrated α-MEM (Gibco, USA) with 0.2 IU/ml FSH (Solarbio Life Science, China), 10% FBS, 10 ng/mL EGF (Abbkine, China) and 1% penicillin-streptomycin solution in an incubator at 5% CO_2_ and 37 °C. The first polar body extruded after in vitro maturation for 16–18 h.

### Immunohistochemistry

Briefly, the ovarian tissue sections were incubated with the following primary antibodies: polyclonal rabbit anti-CD31 (1:200; Abcam, ab124432, British), polyclonal rabbit anti-CD34 (1:200; Abcam, ab185732, British) and polyclonal rabbit anti-FSHR (1:100; Proteintech, Cat No. 22665-1-AP, China). After washing in PBS, the sections were incubated with a secondary biotinylated goat anti-rabbit IgG antibody (1:1 000; Abcam, ab64256, UK).

### Hormone level assay

The peripheral blood of the three transplantation groups was obtained by the eye posterior orbital venous plexus of the mice in the dioestrus stage of the oestrus cycle. We stored the samples at RT for 1 h and collected the serum after centrifugation at 1000 × *g* for 10 min. Then, the levels of AMH, E_2_, FSH and LH were measured according to the manufacturer’s instructions (Cloud-Clone Corp, China).

### Western blotting

Proteins (40 μg) were separated by SDS-PAGE and transferred to 0.45 μm PVDF membranes. Nonspecific binding was blocked with 5% nonfat milk for 1 h at RT. The membranes were hybridized with the primary antibodies anti-p-eNOS (1:500; Abcam, ab215717, UK), anti-eNOS (1:2 000; CST, #32027, USA) and anti-GAPDH (1:10 000; Abcam, ab37168, UK) in TBST (Tris Buffered Saline with Tween® 20) that contained 1% nonfat milk overnight. The membranes were washed with TBST three times, each time for 10 min, followed by incubation with goat anti-rabbit horseradish peroxidase (HRP)-conjugated secondary antibody (1:10 000; ASPEN, AS1107, UK) at 37 °C for 1 h. After washing three washes with TBST, immunoreactive bands were visualized by an enhanced chemiluminescent system (P1108, Beyotime Institute of Biotechnology, China), and the uncropped and unprocessed scans of the blots was showed in Supplementary Fig. 21. The band intensity was quantified by densitometry using Quantity One 4.6.9 analysis software (Bio-Rad, USA). *GAPDH* was used as an internal reference gene to statistically analyze the expression of the targeted protein.

### Follicle counting

Serial paraffin sections (4 μm thick) were created from the ovarian tissues on the contralateral and transplant sides. For continuous paraffin sectioning, one out of every five intervals was taken for staining with H&E. All stages of follicles (primordial, primary, secondary, antral follicles) were detected and classified. The area of ovarian tissue and number of follicles from each ovary were calculated and compared between each group.

### TUNEL assay

A terminal deoxynucleotidyl transferase-mediated dUTP nick end labeling (TUNEL) assay was performed using riboAPO™ One-Step TUNEL Apoptosis Kit (Green) (RiboBio, Guangzhou, China). For 4 μm-thick paraffin sections, the slices were dewaxed and rehydrated according to standard protocols, and the sections were then rinsed twice with distilled water for 2 min. After the addition of 100 μL Protein K working solution (l20 μg/mL) to the paraffin sections and incubation for 10 min at 37 °C, the slices were rinsed three times with distilled water, followed by fixation and rinsing. The samples, as well as the positive/negative control prepared according to the instructions, were treated by the addition of 50 μL TDT reaction mixture that contained TdT enzyme (replaced with 1×TDT enzyme buffer for the negative-control group), fluorescein-dUTP mixture and 1×TDT enzyme buffer, incubated at 37 °C for 2 h in a humidified atmosphere, and 100 μL 2×SSC was added for 15 min to terminate the reaction at RT, followed by rinsing three times with PBS. Finally, the samples were counterstained with DAPI and analyzed under a fluorescence microscope.

### Statistical analysis

Each experiment was repeated more than three times with consistent results, and the data (presented as the mean ± SD) and statistical significance were analyzed by using SPSS Statistics 17.0 and GraphPad Prism 8 (GraphPad Software Inc.). Normally distributed numerical variance was performed by one or two-way ANOVA with homogeneity of variance, two-sided Student’s t test, and chi square tests (χ2 test) were employed for the differences between two or more rates. Statistical significance was assumed at *P* < 0.05. Non- significant differences were labeled in figures as “ns”.

### Reporting summary

Further information on research design is available in the Nature Research Reporting Summary linked to this article.

## Supplementary information

Supplementary Information-revised

REPORTING SUMMARY

## Data Availability

All data supporting the findings of this work are available within the Supplementary Materials, with all detailed data included in Supplementary Tables 1–3 and Supplementary Figs. 1–21. The datasets generated and analyzed during the current study are available from the corresponding authors upon request.
