# Supplementary material for: Multi-biofunctional graphene oxide-enhanced poly-L-lactic acid composite nanofiber scaffolds for ovarian function recovery of transplanted-tissue
Source: NPJ Regen Med. 2022 Sep 16;7:52. doi: 10.1038/s41536-022-00236-5 (PMC9481528; doi:10.1038/s41536-022-00236-5)
Supplement: Supplementary file 1 — Supplementary Information-revised [file 41536_2022_236_MOESM1_ESM.pdf]

SUPPLEMENTARY INFORMATION FOR

Multi-biofunctional graphene oxide-enhanced poly-*L*-lactic acid composite nanofiber scaffolds for ovarian function recovery of transplanted-tissue

Liang Yan [1, 2, 7 †], Lingjuan Wang [1, 3 †], Jiachen Wu [3 †], Yuanzheng Wu [2], Xianyu Zhu [2], Qiaojuan Mei [1], Yinhua Song [3], Yang Liu [3], Ling Zhang [1], Jihui Ai [3], Kezhen Li [3], Guangming Qing [4], Yong Zhang [5 *], Xianjin Xiao [1 *], Yuliang Zhao [2, 6, 7 *], Wenpei Xiang [1 *]

*Corresponding authors. Institute of Reproductive Health, Center of Reproductive Medicine, Tongji Medical College, Huazhong University of Science and Technology. Wuhan 430030, China. Tel: 86-13886166929; Fax: 86-27-83692605. E-mail: wpxiang2010@gmail.com; zhaoyl@nanoctr.cn; xiaoxianjin@hust.edu.cn; mailzhangyong@126.com.

SUPPLEMENTARY TEXT

Supplementary Figure 1 | Characterization of GO nanosheets.

Supplementary Figure 2 | AFM images and R_a roughness of GO nanosheets adsorbed with or without PLLA.

Supplementary Figure 3 | Thickness, roughness of GO/PLLA nanofiber scaffolds.

Supplementary Figure 4 | Schematic view shows the approach for the preparation of GO/PLLA nanofiber scaffolds.

Supplementary Figure 5 | Optical images of as-made GO/PLLA nanofiber scaffolds with the concentration of GO ranging from 0.0 to 4.0wt% (From left to right).

Supplementary Figure 6 | Raman spectra of GO/PLLA nanofiber scaffolds.

Supplementary Figure 7 | Mechanical properties of GO/PLLA nanofiber scaffolds.

Supplementary Figure 8 | Distribution of the diameter of nanofibers.

Supplementary Figure 9 | Contact angles, and porosity of GO/PLLA nanofiber scaffolds.

Supplementary Figure 10 | SEM images of GO/PLLA nanofiber scaffolds degraded in DI water.

Supplementary Figure 11 | SEM images of GO/PLLA nanofiber scaffolds degraded in PBS.

Supplementary Figure 12 | SEM images of GO/PLLA nanofiber scaffolds degraded in DMEM.

Supplementary Figure 13 | SEM images of GO/PLLA nanofiber scaffolds degraded in DMEM supplemented with FBS.

Supplementary Figure 14 | Raman spectra of 0.0wt% GO/PLLA nanofiber scaffold degraded in different media.

Supplementary Figure 15 | Raman spectra of 0.5wt% GO/PLLA nanofiber scaffold degraded in different media.

Supplementary Figure 16 | Raman spectra of 1.0wt% GO/PLLA nanofiber scaffold degraded in different media.

Supplementary Figure 17 | Raman spectra of 4.0wt% GO/PLLA nanofiber scaffold degraded in different media.

Supplementary Figure 18 | Weight loss of GO/PLLA nanofiber scaffolds treated with different media at 28 days.

Supplementary Figure 19 | SEM images of GO/PLLA nanofiber scaffolds for the evaluation of degradation behavior *in vivo*.

Supplementary Figure 20 | Changes in ovarian function after cisplatin injection.

Supplementary Figure 21 | The source data of western blots in Figure 7

Supplementary Table 1 | Serum hormone levels in the control and POI groups.

Supplementary Table 2 | Survival of transplanted ovarian tissue.

Supplementary Table 3 | The obtained oocytes number and mature oocytes (MII) *in vitro*.

SUPPLEMENTARY MATERIALS

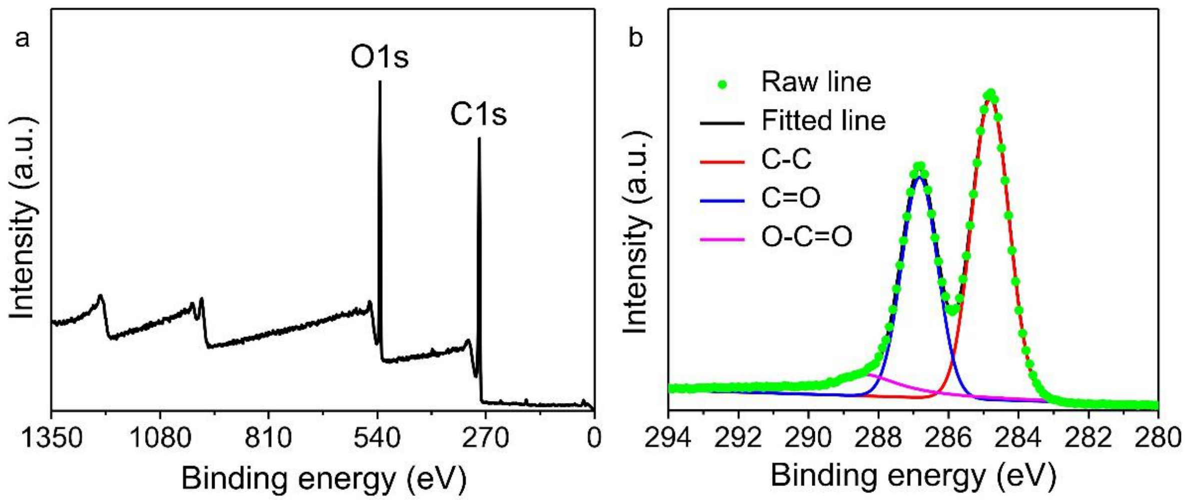

Supplementary Figure 1 | Characterization of GO nanosheets. (a) Full survey XPS spectrum of GO nanosheets. (b) XPS spectrum of C 1s. The peak located at 284.79 eV can be contributed to the sp^2 peak of the C 1s, suggesting the presence of hydrophobic aromatic domain capable of attaching hydrophobic moieties via hydrophobic interactions. Meanwhile, the peaks centered at 286.85 eV and 288.55 eV are related to carbonyl (-C=O) and carboxyl (-O-C=O) groups, respectively.

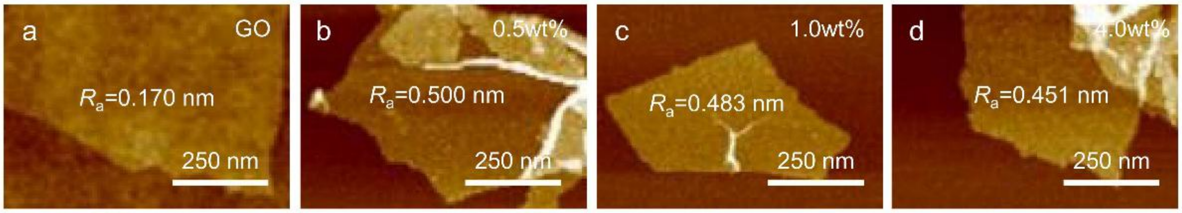

Supplementary Figure 2 | AFM images and R_a roughness of GO nanosheets adsorbed with or without PLLA. The increase in the R_a roughness demonstrates the large density of PLLA adsorbed on the surface of GO nanosheets.

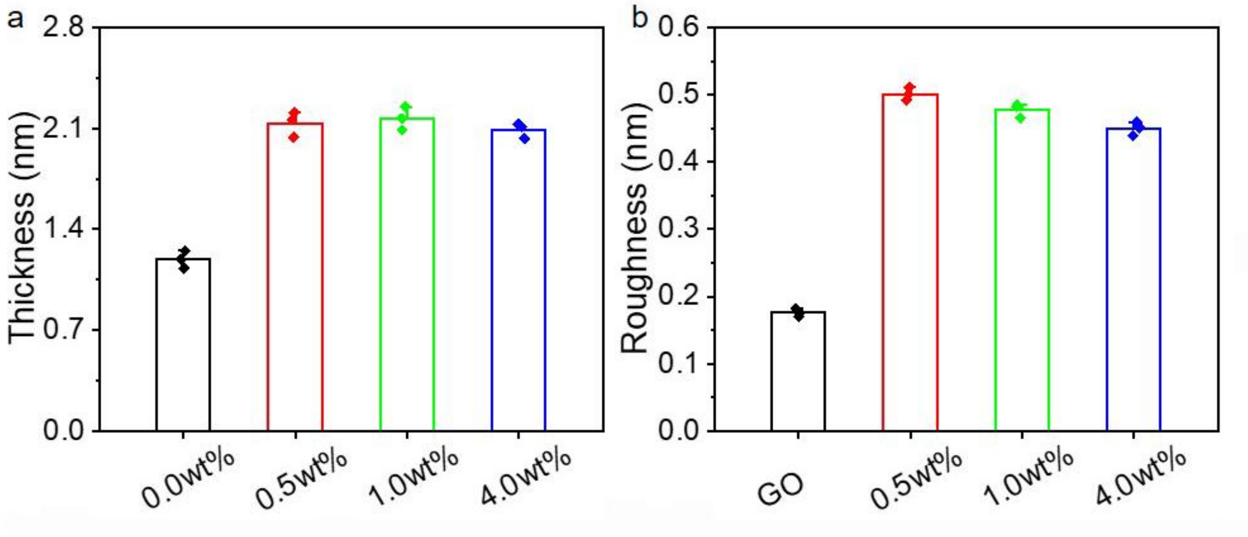

Supplementary Figure 3 | Thickness (a) and roughness (b) of GO/PLLA nanofiber scaffolds.

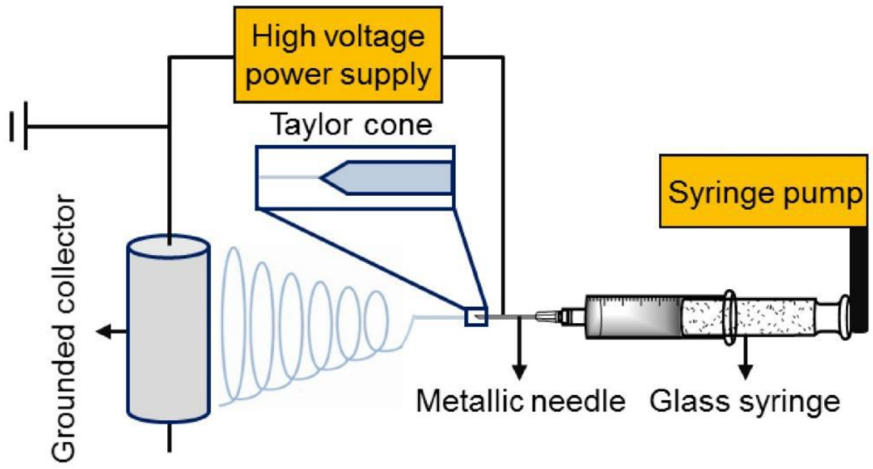

Supplementary Figure 4 | Schematic view shows the approach for the preparation of GO/PLLA nanofiber scaffolds.

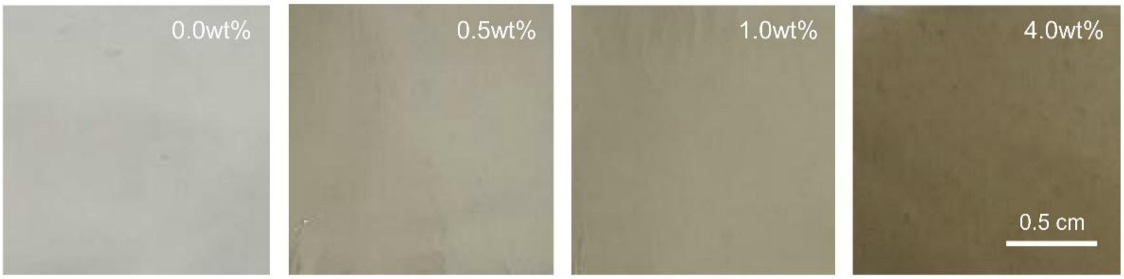

Supplementary Figure 5 | Optical images of as-made GO/PLLA nanofiber scaffolds with the concentration of GO ranging from 0.0 to 4.0wt% (from left to right).

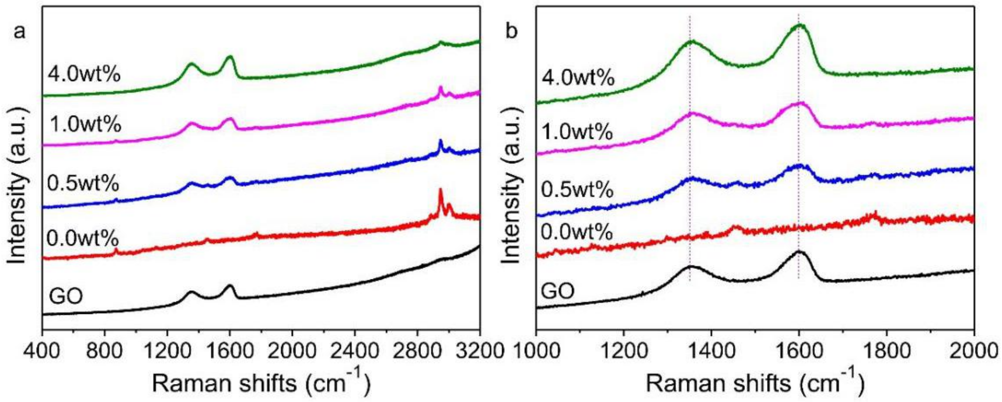

Supplementary Figure 6 | Raman spectra of GO/PLLA nanofiber scaffolds. As shown in Figure S6a, the spectrum of GO nanosheets exhibits two characteristic peaks located at 1350.19 cm^{-1} and 1597.55 cm^{-1}, which correspond to D band resulted from defects and disorders and G band related to aromatic rings, respectively.

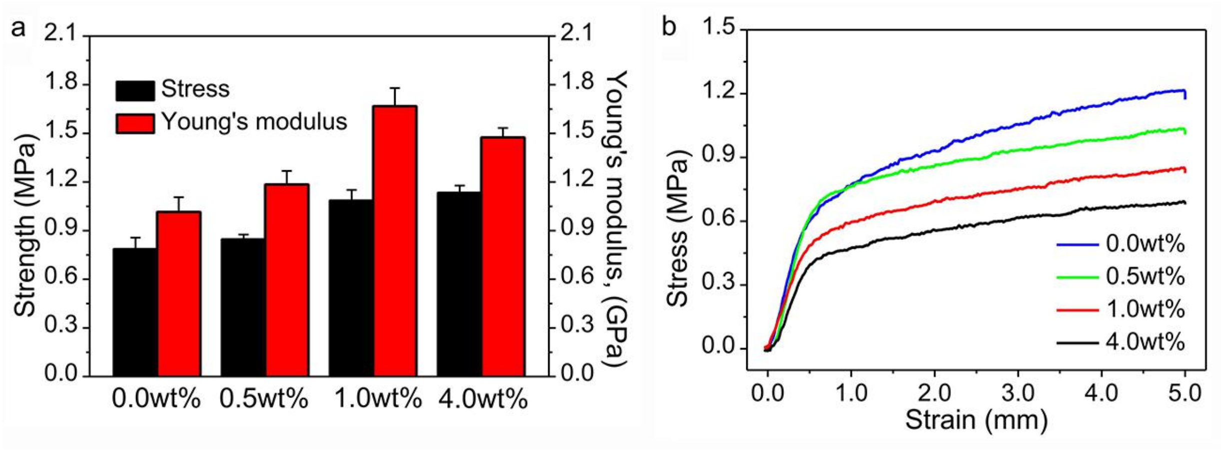

Supplementary Figure 7 | Mechanical properties of GO/PLLA nanofiber scaffolds. (a) The trends of the Young's modulus and strength for GO/PLLA nanofiber scaffolds. (b) The tensile stress-strain curves for GO/PLLA nanofiber scaffolds.

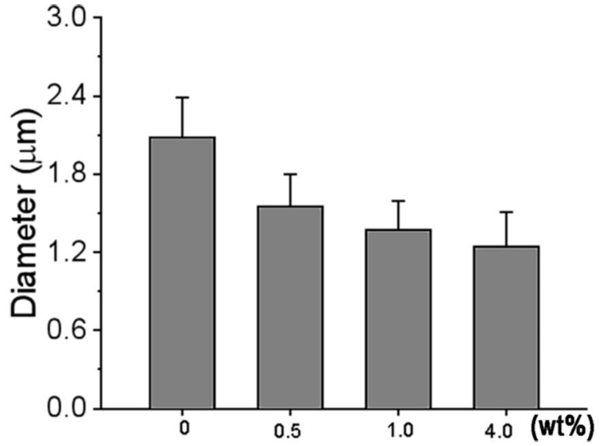

Supplementary Figure 8 | Distribution of the diameter of nanofibers.

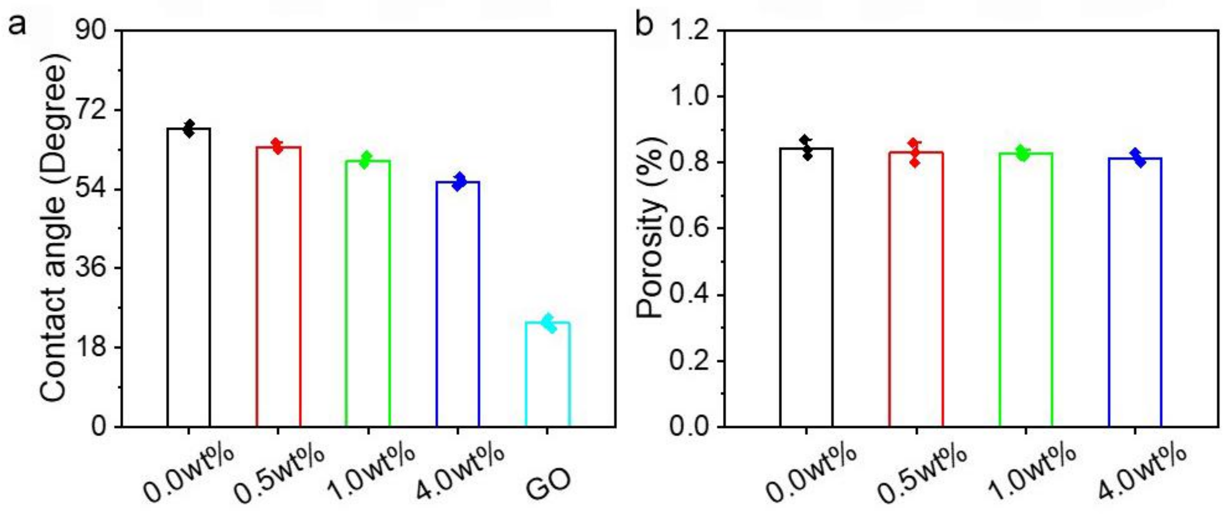

Supplementary Figure 9 | Contact angles (a) and porosity (b) of GO/PLLA nanofiber scaffolds.

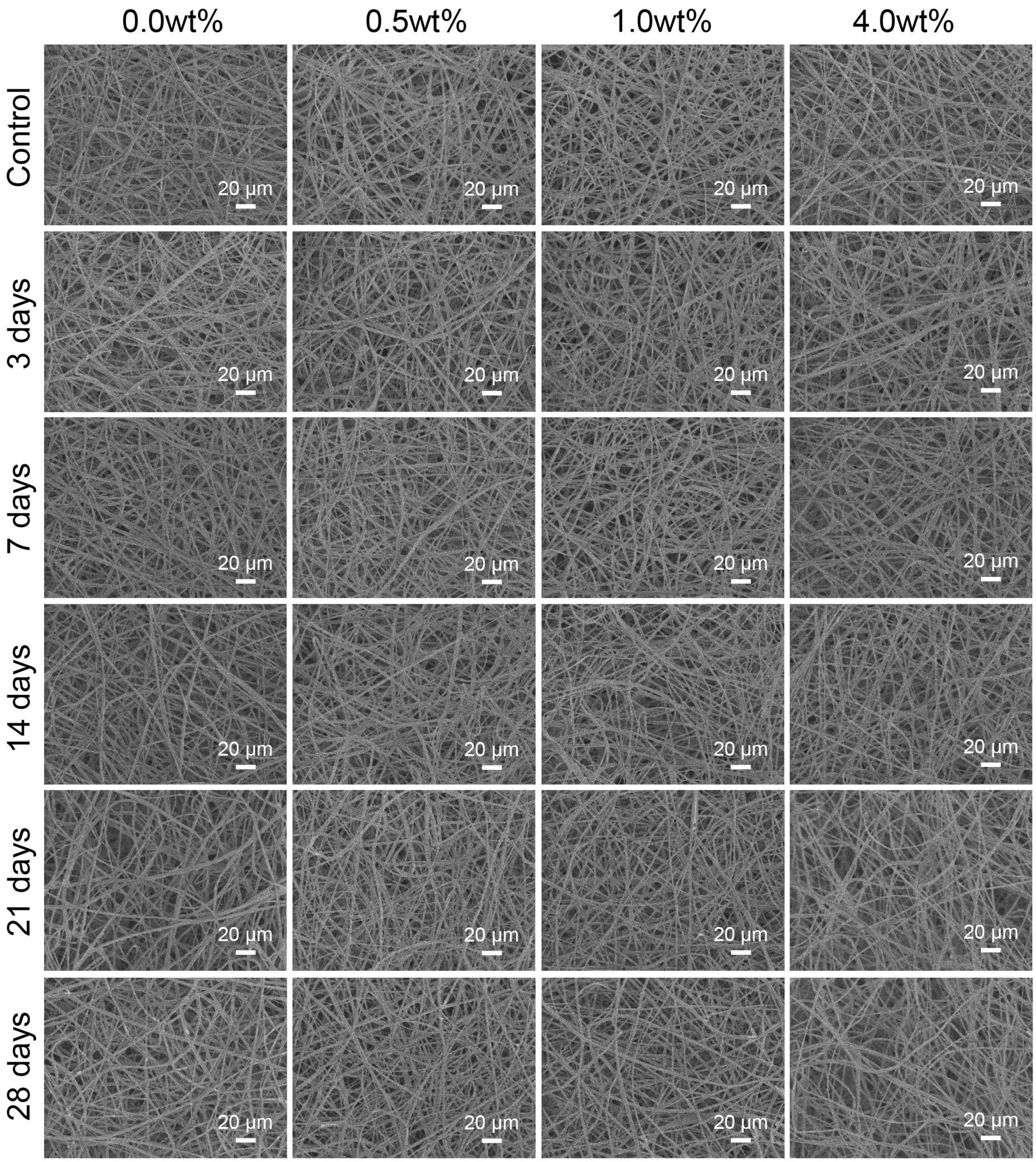

Supplementary Figure 10 | SEM images of GO/PLLA nanofiber scaffolds degraded in DI water for 0, 3, 7, 14, 21, and 28 days.

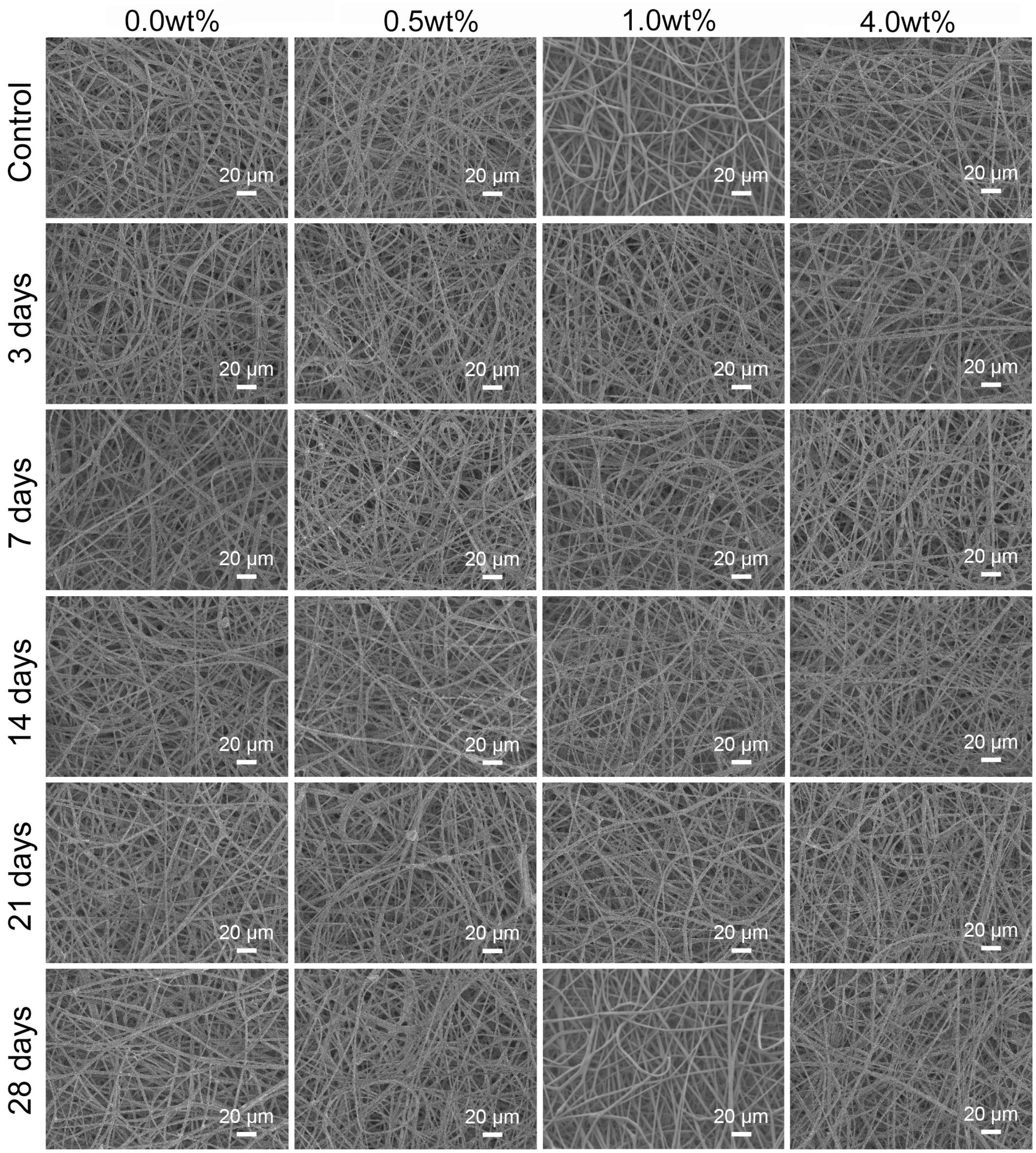

Supplementary Figure 11 | SEM images of GO/PLLA nanofiber scaffolds degraded in PBS for 0, 3, 7, 14, 21, and 28 days.

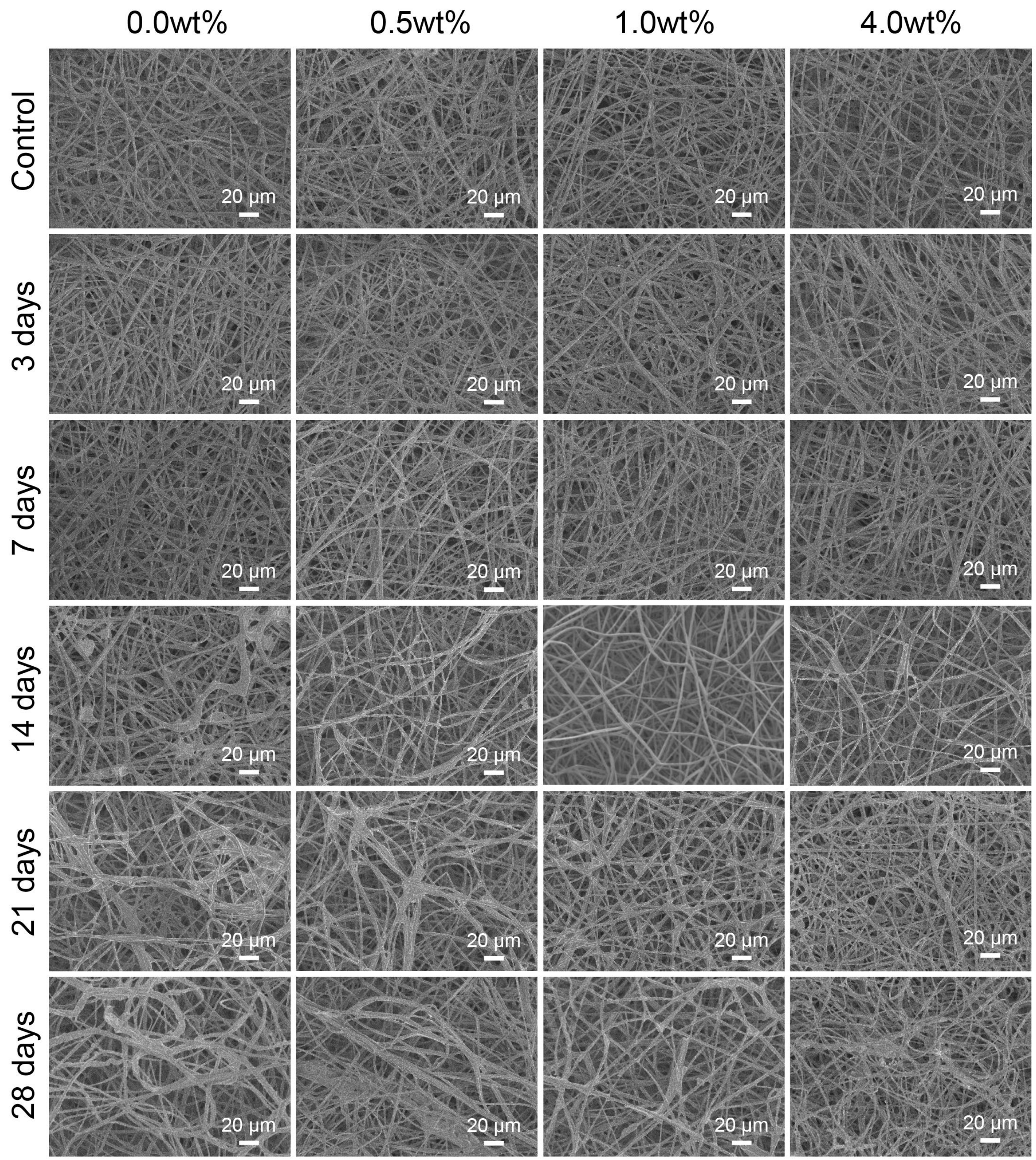

Supplementary Figure 12 | SEM images of GO/PLLA nanofiber scaffolds degraded in DMEM for 0, 3, 7, 14, 21, and 28 days.

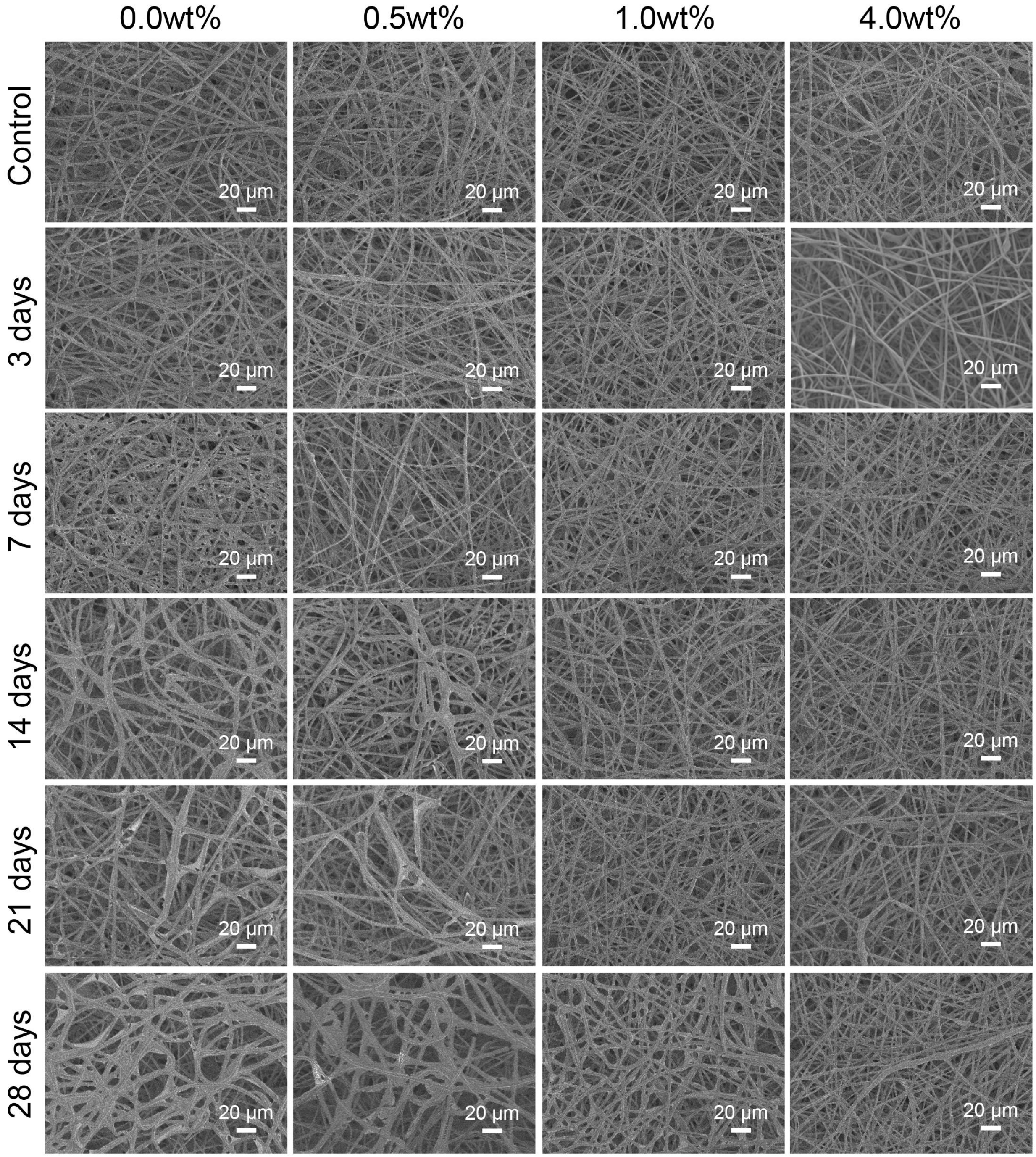

Supplementary Figure 13 | SEM images of GO/PLLA nanofiber scaffolds degraded in DMEM supplemented with FBS for 0, 3, 7, 14, 21, and 28 days.

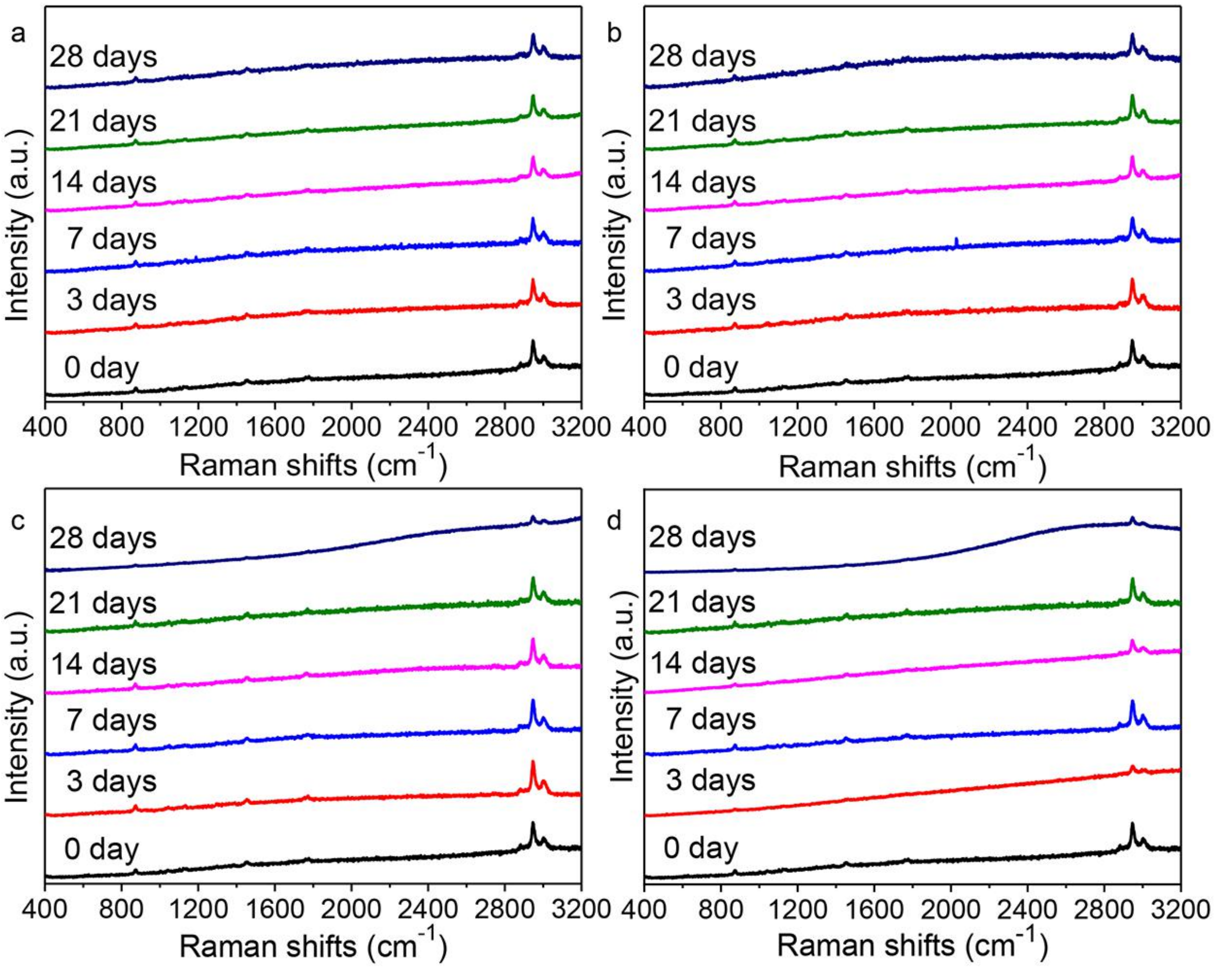

Supplementary Figure 14 | Raman spectra of 0.0wt% GO/PLLA nanofiber scaffold degraded in DI water (a), PBS (b), DMEM (c), and DMEM/FBS (d) for 0, 3, 7, 14, 21, and 28 days.

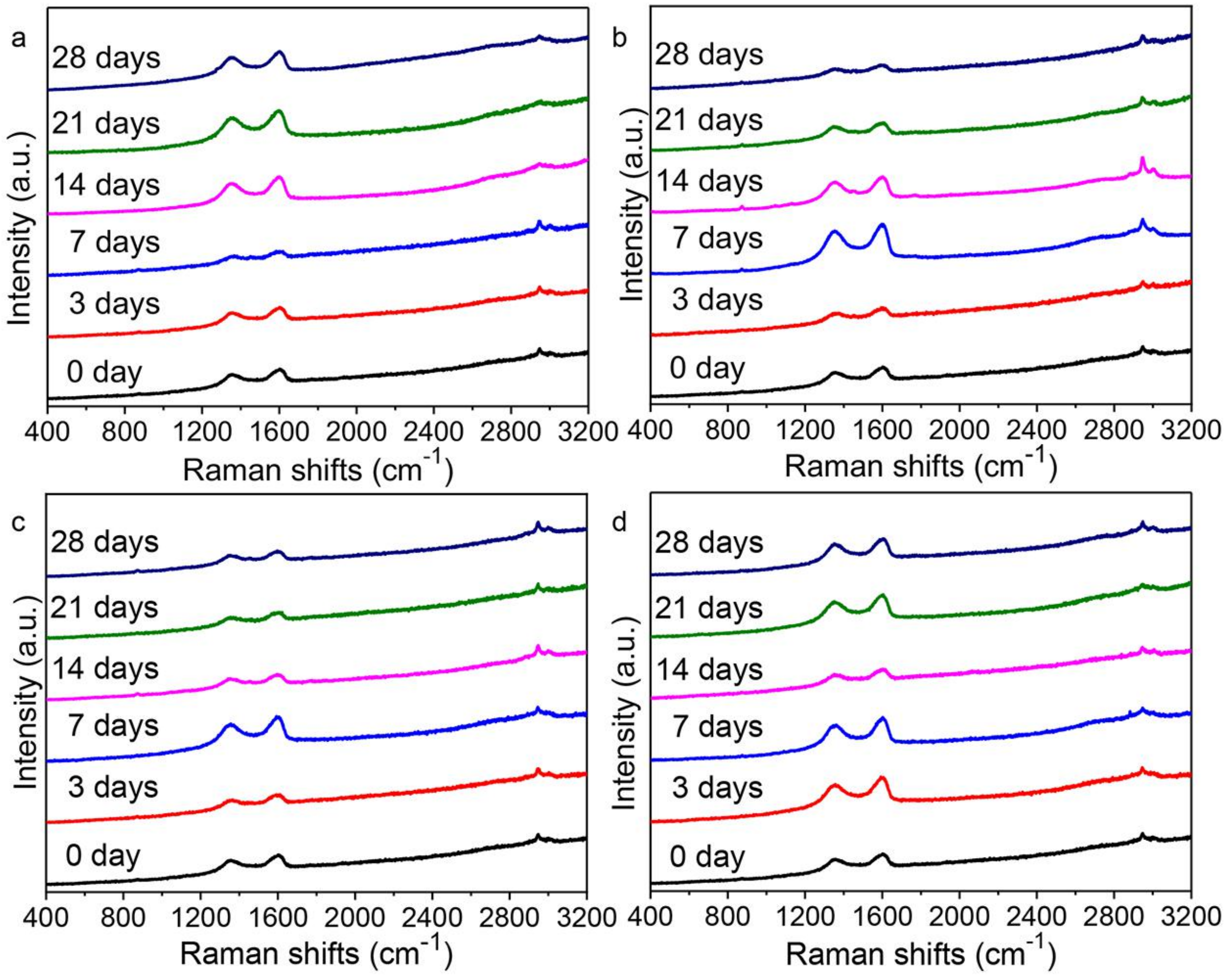

Supplementary Figure 15 | Raman spectra of 0.5wt% GO/PLLA nanofiber scaffold degraded in DI water (a), PBS (b), DMEM (c), and DMEM/FBS (d) for 0, 3, 7, 14, 21, and 28 days.

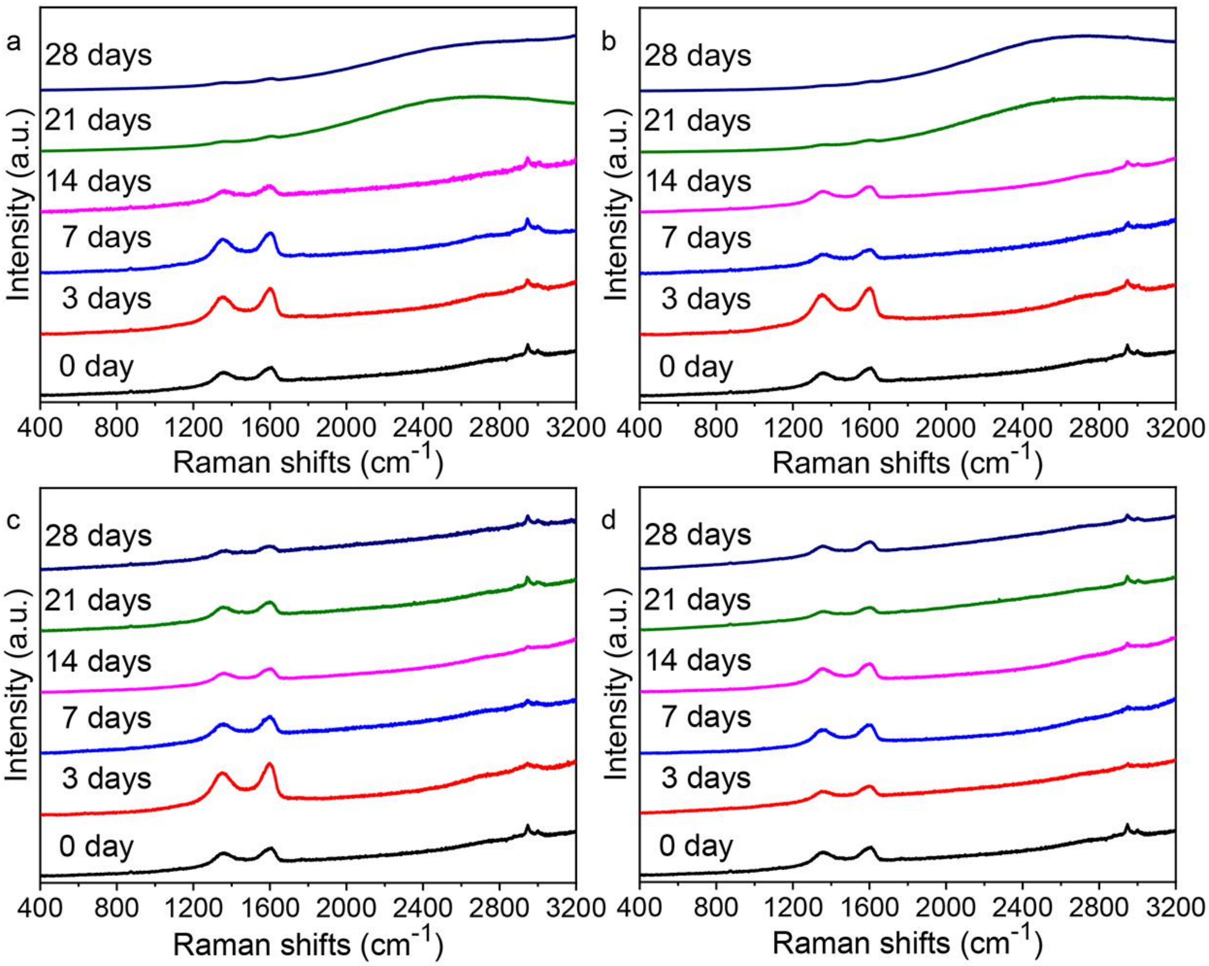

Supplementary Figure 16 | Raman spectra of 1.0wt% GO/PLLA nanofiber scaffold degraded in DI water (a), PBS (b), DMEM (c), and DMEM/FBS (d) for 0, 3, 7, 14, 21, and 28 days.

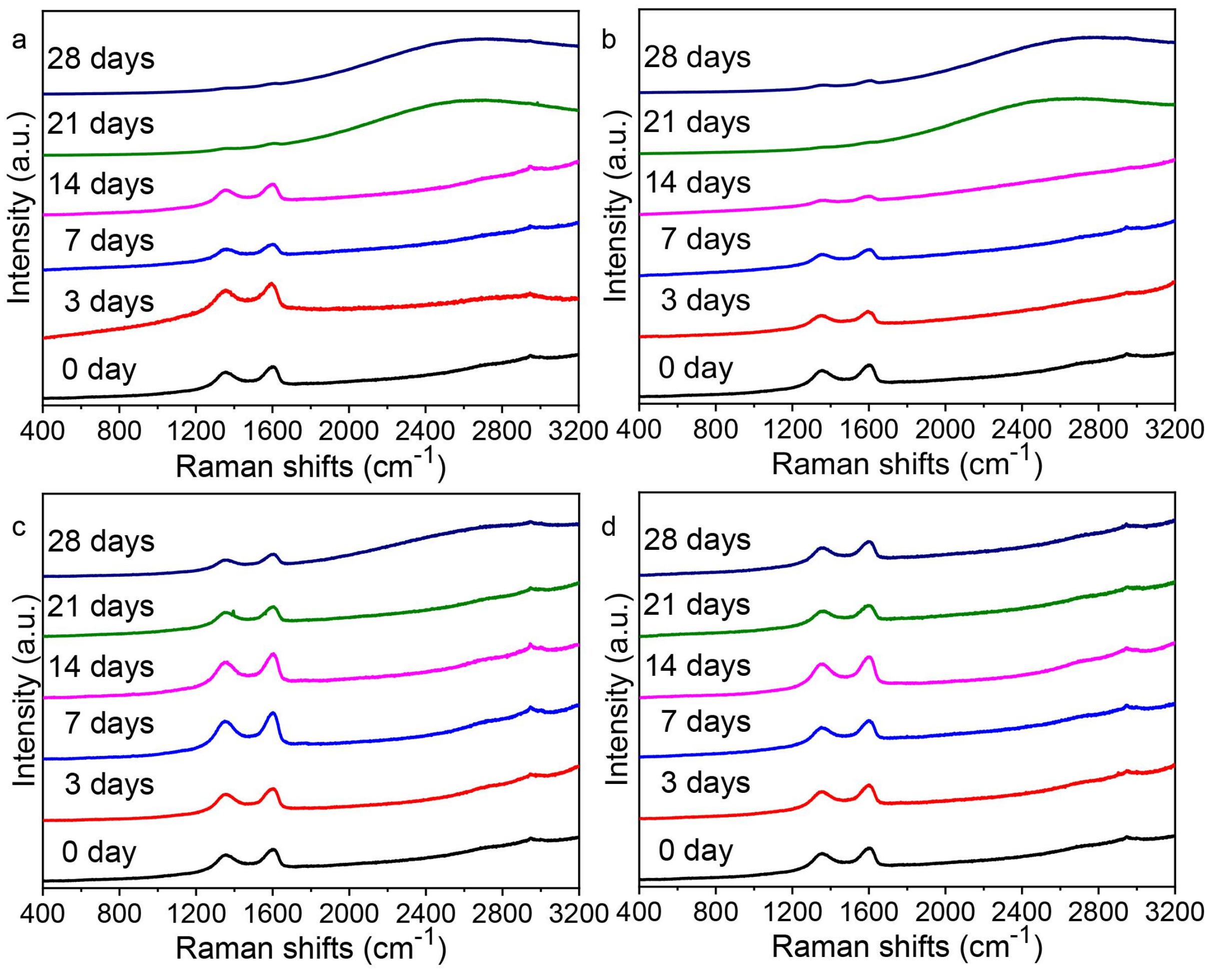

Supplementary Figure 17 | Raman spectra of 4.0wt% GO/PLLA nanofiber scaffold degraded in DI water (a), PBS (b), DMEM (c), and DMEM/FBS (d) for 0, 3, 7, 14, 21, and 28 days. Notably, the Raman Spectra different for samples in different media. The main reason is listed as follows: After treatment with media, chemical species, such as ions and proteins, can interact with GO/PLLA nanofiber scaffolds, largely leading to a shift in the Raman peak and peak shape distortion. Moreover, some chemical species have fluorescence properties, in particular proteins, and can cause an increase in background signal noise, making it difficult to distinguish the Raman signal from the background. Therefore, the Raman spectra may be different for the same sample treated with different media.

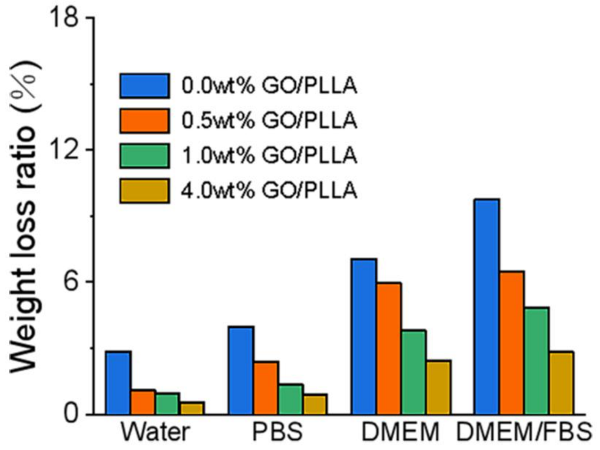

Supplementary Figure 18 | Weight loss of GO/PLLA nanofiber scaffolds treated with different media at 28 days.

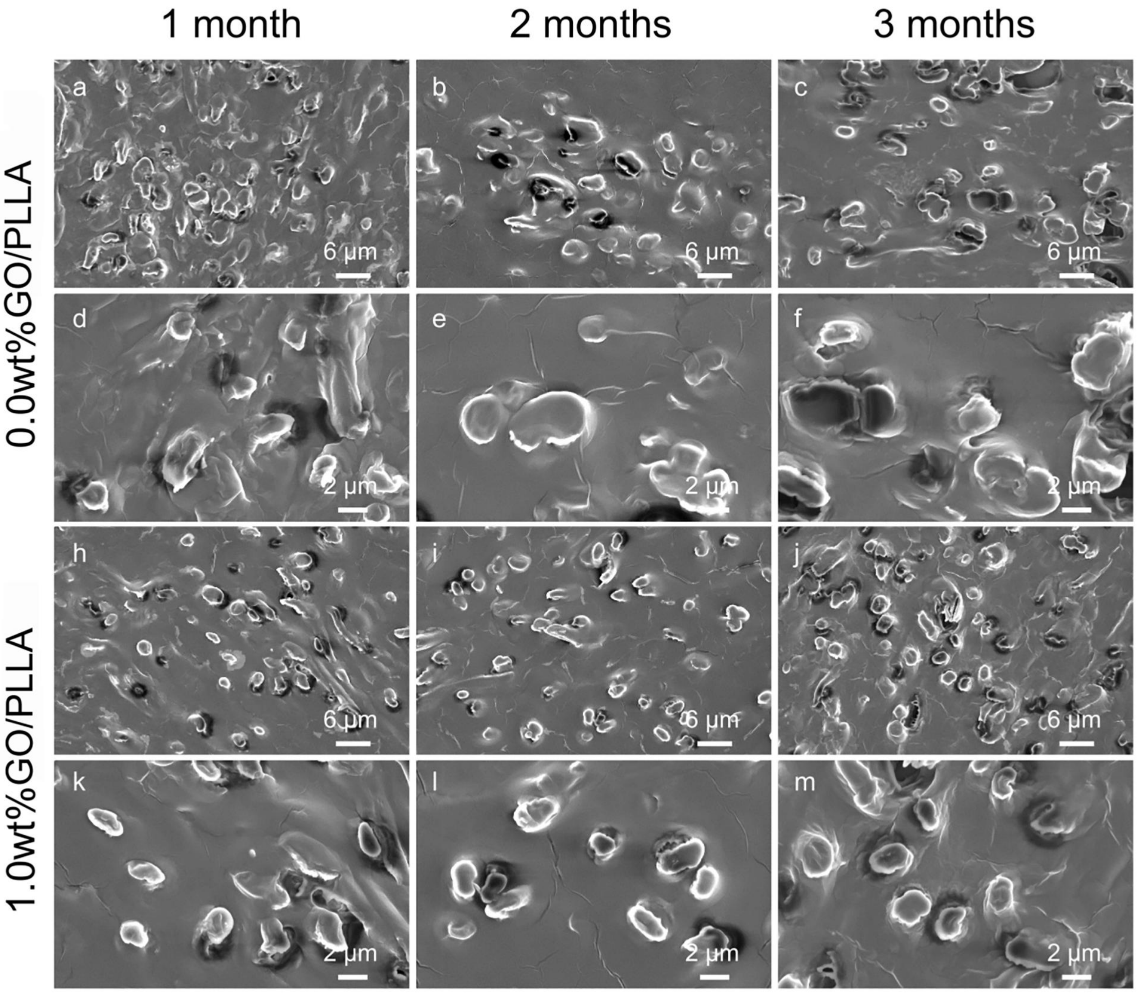

Supplementary Figure 19 | SEM images of GO/PLLA nanofiber scaffolds for the evaluation of degradation behavior *in vivo*.

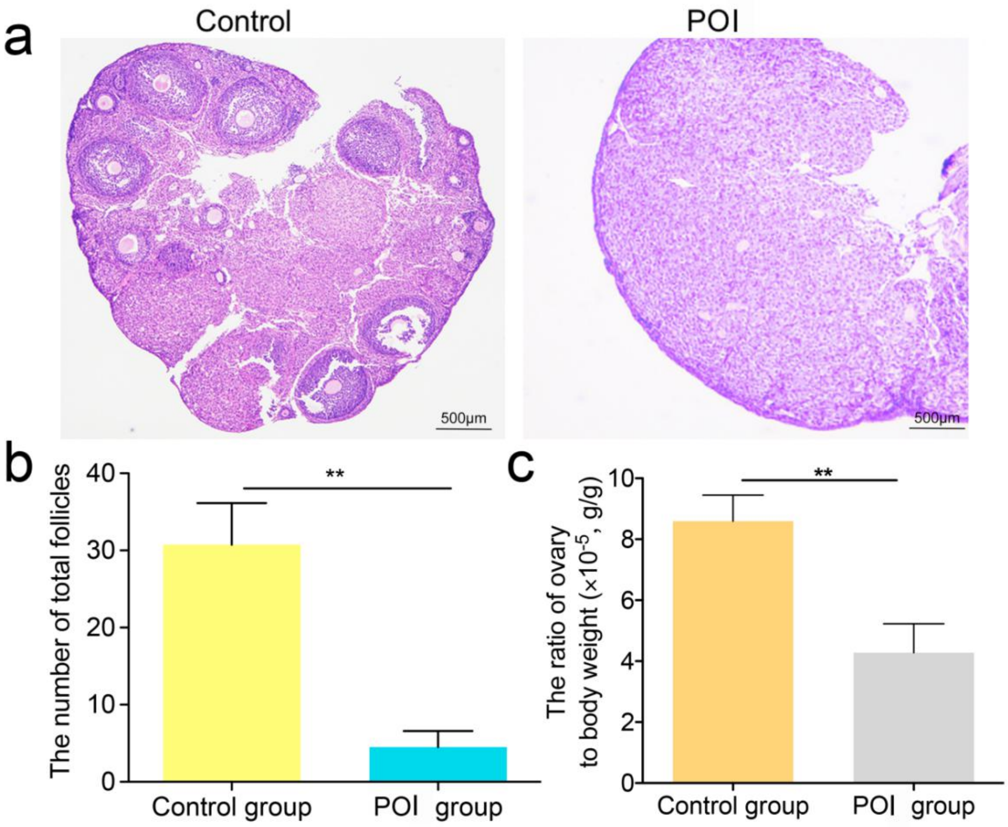

Supplementary Figure 20 | Changes in ovarian function after cisplatin injection. (a) Representative images of HE staining for histopathological observation of ovaries. (b) The number of total follicles in control and POI groups. (c) The ratio of ovary to body weight (g/g) in the control and POI groups. Statistical significance levels are set at $^{**}P < 0.01$ by two-tailed Student's t-test. Data are presented as mean ± SD, scale bar = 500 μm.

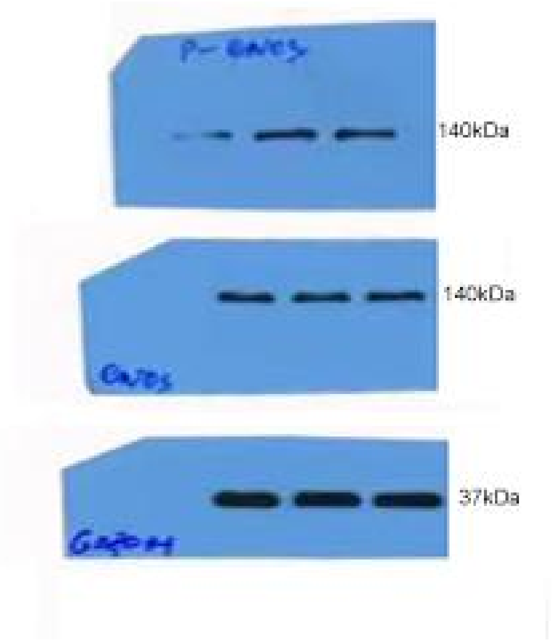

Supplementary Figure 21 | The source data of western blots in Figure 7

Supplementary Table 1 | Serum hormone levels in the control and POI groups.

Hormone levels	Control group	POI group
FSH (ng/ml)	20.34±1.48	43.46±3.40**
LH (ng/ml)	3.797±0.54	12.38±0.54**
E_2 (pg/ml)	46.34±2.28	14.43±1.54**
AMH (ng/ml)	4.22±0.22	0.51±0.15**

**$P < 0.01$ (POI *vs* Control group)

Supplementary Table 2 | Survival of transplanted ovarian tissue.

Groups	No. of mice (n/n)				The ovary survival rate (%)			
	1 month	2 months	3 months	total	1 month	2 months	3 months	total
Ovary	0/5	2/4	2/5	4/14	0.0	50.0	40.0	28.6
Ovary+0.0wt% GO/PLLA	2/4	2/4	3/5	7/13	50.0	50.0	60.0	53.8*
Ovary+1.0wt% GO/PLLA	5/5	3/4	5/6	13/15	100.0	75.0	83.3	86.7**

**$P < 0.01$ vs. Ovary+0.0wt% GO/PLLA group and Ovary group

*$P < 0.05$ vs. Ovary group

Supplementary Table 3 | The obtained oocytes number and mature oocytes (MII) *in vitro*.

Groups	Total oocytes/3 mice（n）	MⅡ stage (n)	Mature percentage (%)
Normal-Control	12	10	83.3
Ovary	2	0	0
Ovary+0.0wt% GO/PLLA	6	3	50
Ovary+1.0wt% GO/PLLA	7	6	85.7
