# Supplementary material for: Multi-biofunctional graphene oxide-enhanced poly-L-lactic acid composite nanofiber scaffolds for ovarian function recovery of transplanted-tissue
Source: NPJ Regen Med. 2022 Sep 16;7:52. doi: 10.1038/s41536-022-00236-5 (PMC9481528; doi:10.1038/s41536-022-00236-5)
Supplement: Supplementary file 2 — REPORTING SUMMARY [file 41536_2022_236_MOESM2_ESM.pdf]

nature research

Corresponding author(s): DBPR NPJREGENMED-00908R1

Reporting Summary

Nature Research wishes to improve the reproducibility of the work that we publish. This form provides structure for consistency and transparency in reporting. For further information on Nature Research policies, see Authors & Referees and the Editorial Policy Checklist.

Statistical parameters

When statistical analyses are reported, confirm that the following items are present in the relevant location (e.g. figure legend, table legend, main text, or Methods section).

n/a	Confirmed	
☐	☒	The exact sample size (n) for each experimental group/condition, given as a discrete number and unit of measurement
☐	☒	An indication of whether measurements were taken from distinct samples or whether the same sample was measured repeatedly
☐	☒	The statistical test(s) used AND whether they are one- or two-sided<br>*Only common tests should be described solely by name; describe more complex techniques in the Methods section.*
☐	☒	A description of all covariates tested
☐	☒	A description of any assumptions or corrections, such as tests of normality and adjustment for multiple comparisons
☐	☒	A full description of the statistics including central tendency (e.g. means) or other basic estimates (e.g. regression coefficient) AND variation (e.g. standard deviation) or associated estimates of uncertainty (e.g. confidence intervals)
☐	☒	For null hypothesis testing, the test statistic (e.g. F, t, r) with confidence intervals, effect sizes, degrees of freedom and P value noted<br>*Give P values as exact values whenever suitable.*
☒	☐	For Bayesian analysis, information on the choice of priors and Markov chain Monte Carlo settings
☐	☒	For hierarchical and complex designs, identification of the appropriate level for tests and full reporting of outcomes
☐	☒	Estimates of effect sizes (e.g. Cohen's d, Pearson's r), indicating how they were calculated
☐	☒	Clearly defined error bars<br>*State explicitly what error bars represent (e.g. SD, SE, CI)*

Our web collection on statistics for biologists may be useful.

Software and code

Policy information about availability of computer code

Data collection	No software was used.
Data analysis	No software was used.

For manuscripts utilizing custom algorithms or software that are central to the research but not yet described in published literature, software must be made available to editors/reviewers upon request. We strongly encourage code deposition in a community repository (e.g. GitHub). See the Nature Research guidelines for submitting code & software for further information.

Data

Policy information about availability of data

All manuscripts must include a data availability statement. This statement should provide the following information, where applicable:

- Accession codes, unique identifiers, or web links for publicly available datasets
- A list of figures that have associated raw data
- A description of any restrictions on data availability

A list of figures:
Supplementary Figure 1 | Characterization of GO nanosheets.
Supplementary Figure 2 | AFM images and Ra roughness of GO nanosheets adsorbed with or without PLLA.

Supplementary Figure 3 | Thickness, roughness of GO/PLLA nanofiber scaffolds.
Supplementary Figure 4 | Schematic view shows the approach for the preparation of GO/PLLA nanofiber scaffolds.
Supplementary Figure 5 | Optical images of as-made GO/PLLA nanofiber scaffolds with the concentration of GO ranging from 0.0 to 4.0wt% (From left to right).
Supplementary Figure 6 | Raman spectra of GO/PLLA nanofiber scaffolds.
Supplementary Figure 7 | Mechanical properties of GO/PLLA nanofiber scaffolds.
Supplementary Figure 8 | Distribution of the diameter of nanofibers.
Supplementary Figure 9 | Contact angles, and porosity of GO/PLLA nanofiber scaffolds.
Supplementary Figure 10 | SEM images of GO/PLLA nanofiber scaffolds degraded in DI water.
Supplementary Figure 11 | SEM images of GO/PLLA nanofiber scaffolds degraded in PBS.
Supplementary Figure 12 | SEM images of GO/PLLA nanofiber scaffolds degraded in DMEM.
Supplementary Figure 13 | SEM images of GO/PLLA nanofiber scaffolds degraded in DMEM supplemented with FBS.
Supplementary Figure 14 | Raman spectra of 0.0wt% GO/PLLA nanofiber scaffold degraded in different media.
Supplementary Figure 15 | Raman spectra of 0.5wt% GO/PLLA nanofiber scaffold degraded in different media.
Supplementary Figure 16 | Raman spectra of 1.0wt% GO/PLLA nanofiber scaffold degraded in different media.
Supplementary Figure 17 | Raman spectra of 4.0wt% GO/PLLA nanofiber scaffold degraded in different media.
Supplementary Figure 18 | Weight loss of GO/PLLA nanofiber scaffolds treated with different media at 28 days.
Supplementary Figure 19 | SEM images of GO/PLLA nanofiber scaffolds for the evaluation of degradation behavior in vivo.
Supplementary Figure 20 | Changes in ovarian function after cisplatin injection.
Supplementary Figure 21 | The source data of western blots in Figure 7
Supplementary Table 1 | Serum hormone levels in the control and POI groups.
Supplementary Table 2 | Survival of transplanted ovarian tissue.
Supplementary Table 3 | The obtained oocytes number and mature oocytes (MII) in vitro.

Field-specific reporting

Please select the best fit for your research. If you are not sure, read the appropriate sections before making your selection.

☒ Life sciences ☐ Behavioural & social sciences

For a reference copy of the document with all sections, see nature.com/authors/policies/ReportingSummary-flat.pdf

Life sciences

Study design

All studies must disclose on these points even when the disclosure is negative.

Sample size	Wild-type C57BL/6 female mice (8 weeks) were randomly divided into two groups: the control group (n=10) and the POI group (n=90).
Data exclusions	No data were excluded from the analyses.
Replication	All attempts at replication were successful.
Randomization	Wild-type C57BL/6 female mice (8 weeks) were randomly divided into two groups: the control group (n=10) and the POI group (n=90).
Blinding	The investigators were not blinded to group collection during data collection and analysis.

Materials & experimental systems

Policy information about availability of materials

n/a	Involved in the study
☐	☒ Unique materials
☐	☒ Antibodies
☒	☐ Eukaryotic cell lines
☐	☒ Research animals
☒	☐ Human research participants

Unique materials

Obtaining unique materials	There are no restrictions on the availability of unique materials.

Antibodies

Antibodies used	ppolyclonal rabbit anti-CD31 (1:200; Abcam, ab124432, British), polyclonal rabbit anti-CD34 (1:200; Abcam, ab185732, British) and polyclonal rabbit anti-FSHR (1:100; Proteintech, Cat No. 22665-1-AP, China), the primary antibodies anti-p-eNOS (1:500;

	Abcam, ab215717, UK), anti-eNOS (1:2 000; CST, #32027, USA) and anti-GAPDH (1:10 000; Abcam, ab37168, UK). secondary biotinylated goat anti-rabbit IgG antibody (1:1 000; Abcam, ab64256, UK); goat anti-rabbit horseradish peroxidase (HRP)-conjugated secondary antibody (1:10 000; ASPEN, AS1107, UK).
Validation	Data provided in the manuscript.

Research animals

Policy information about studies involving animals; ARRIVE guidelines recommended for reporting animal research

Animals/animal-derived materials	Female C57BL/6 mice (8 weeks old, 19~21 g)

Method-specific reporting

n/a	Involved in the study
☒	☐ ChIP-seq
☒	☐ Flow cytometry
☒	☐ Magnetic resonance imaging
